# Structures of CD6 and Its Ligand CD166 Give Insight into Their Interaction

**DOI:** 10.1016/j.str.2015.05.019

**Published:** 2015-08-04

**Authors:** Paul E. Chappell, Lee I. Garner, Jun Yan, Clive Metcalfe, Deborah Hatherley, Steven Johnson, Carol V. Robinson, Susan M. Lea, Marion H. Brown

**Affiliations:** 1 Sir William Dunn School of Pathology, University of Oxford, South Parks Road, Oxford OX1 3RE, UK; 2 Department of Chemistry, Physical and Theoretical Chemistry Laboratory, University of Oxford, South Parks Road, Oxford OX1 3RE, UK

## Abstract

CD6 is a transmembrane protein with an extracellular region containing three scavenger receptor cysteine rich (SRCR) domains. The membrane proximal domain of CD6 binds the N-terminal immunoglobulin superfamily (IgSF) domain of another cell surface receptor, CD166, which also engages in homophilic interactions. CD6 expression is mainly restricted to T cells, and the interaction between CD6 and CD166 regulates T-cell activation. We have solved the X-ray crystal structures of the three SRCR domains of CD6 and two N-terminal domains of CD166. This first structure of consecutive SRCR domains reveals a nonlinear organization. We characterized the binding sites on CD6 and CD166 and showed that a SNP in CD6 causes glycosylation that hinders the CD6/CD166 interaction. Native mass spectrometry analysis showed that there is competition between the heterophilic and homophilic interactions. These data give insight into how interactions of consecutive SRCR domains are perturbed by SNPs and potential therapeutic reagents.

## Highlights

• First structure of consecutive scavenger receptor cysteine rich domains in CD6
• Structure of the two N-terminal domains of CD166 which is the ligand for CD6
• Mapping binding sites on CD6 and CD166
• Insight into how CD6 and its interactions are perturbed by polymorphisms and mAbs

## Graphical Abstract

**Figure:**
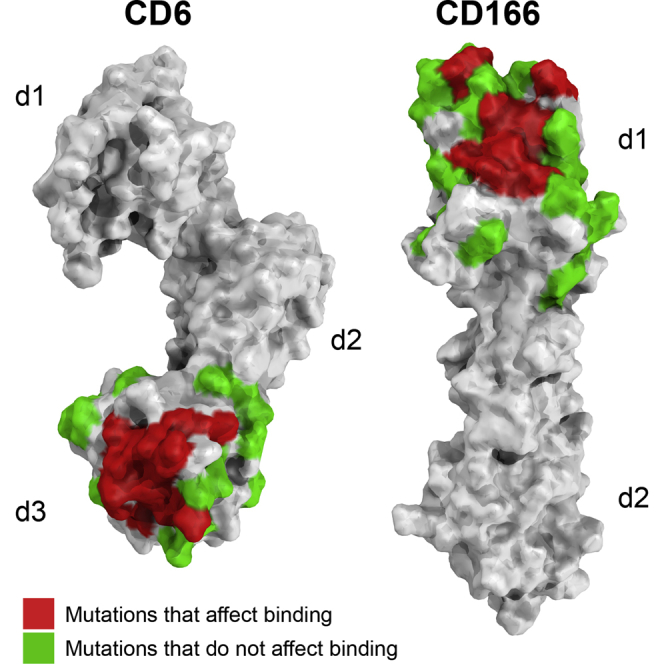

## Teaser

Chappell et al. present structures of the T-cell surface receptor, CD6, the first of consecutive scavenger receptor cysteine rich domains and its ligand, CD166. The structures give insight into how CD6 and its interactions are perturbed by competition between homophilic and heterophilic interactions, SNPs, and mAbs.

## Introduction

**Figure 1:**
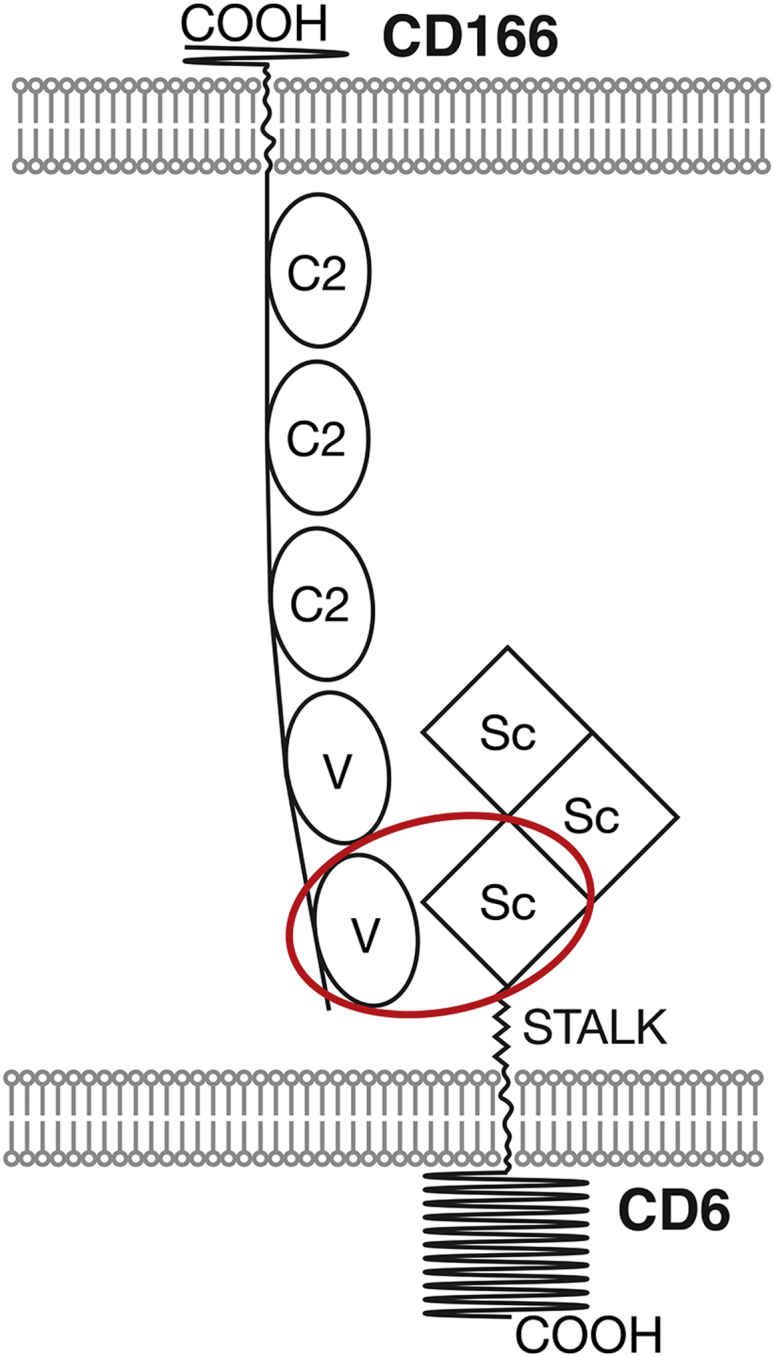
Heterophilic and Homophilic Interactions of CD6 and CD166 between Cells CD6 contains three SRCR domains (Sc, squares) and CD166 contains five IgSF domains (two V and three C2). The membrane proximal domain of CD6 binds the N-terminal immunoglobulin superfamily (IgSF) domain (oval) of CD166.

CD6 is a lymphocyte membrane receptor characterized as being primarily expressed on T cells and some B cells. The extracellular region of CD6 contains three scavenger receptor cysteine rich (SRCR) domains and a membrane proximal stalk (Figure 1). CD6 engages in cell-cell interactions by binding to the immunoglobulin superfamily (IgSF) cell surface receptor, CD166, otherwise known as activated leukocyte adhesion molecule (ALCAM) (Aruffo et al., 1997; Bowen et al., 1995) (Figure 1). CD166 is expressed more widely than CD6 and is found on both hematopoietic and nonhematopoietic cells (Chitteti et al., 2013). In this *trans* interaction between cells, the membrane proximal SRCR domain of CD6 binds the N-terminal domain of CD166 (Aruffo et al., 1997).

In addition to heterophilic interactions with CD6, CD166 also engages in homophilic interactions in *trans* between apposing cells (Te Riet et al., 2007; van Kempen et al., 2001) (Figure 1). As in binding CD6, homophilic interactions of CD166 depend on the N-terminal domain (Bowen et al., 1996; van Kempen et al., 2001). Although weaker than the heterophilic CD6/CD166 interaction (Hassan et al., 2004; Te Riet et al., 2007), CD166 homophilic interactions have pleiotropic effects and regulate not only normal but also neoplastic cells (Chitteti et al., 2013; Weidle et al., 2010). Soluble fusion proteins of the extracellular regions of CD6 or CD166 inhibit T-cell responses (Gimferrer et al., 2004; Hassan et al., 2004; Zimmerman et al., 2006). A CD6 monoclonal antibody (mAb) against domain 3, characterized biochemically as blocking CD6/CD166 interactions distinguished between effects of heterophilic CD6/CD166 and homophilic CD166/CD166 interactions (Hassan et al., 2006). This mAb inhibited antigen-specific proliferation in a polyclonal population of human blood cells, revealing a costimulatory role for engagement of CD6 by CD166 (Hassan et al., 2006). The inhibitory effect of blocking extracellular engagement of CD6 and CD166 identifies these receptors as potential therapeutic targets for immunosuppression.

Costimulation by CD6 is dependent on phosphorylation of a C-terminal tyrosine motif which is specific for the adaptor protein, SLP-76 (Hassan et al., 2006). CD6 has an extraordinarily long cytoplasmic tail (244 amino acids) and provides an alternative to LAT as a scaffold for assemblies of signaling proteins in T cells (Roncagalli et al., 2014). In contrast, CD166 has a short cytoplasmic region that regulates adhesion through a link with the cytoskeleton (Te Riet et al., 2014). Expression of CD6 raises the threshold of activation, and overall net inhibitory or costimulatory effects depend on the particular immune response being measured (Hassan et al., 2006; Oliveira et al., 2012). The dual effects of CD6 make it difficult to interpret the consequences of SNPs that alter the expression of CD6 and correlate with susceptibility to multiple sclerosis (De Jager et al., 2009; Heap et al., 2010; Kofler et al., 2011; Swaminathan et al., 2013).

It is not yet clear how the multiple domains of CD6 are important for function. In the absence of domain 3, no interaction with CD166 can be detected biochemically (Bowen et al., 1996; Hassan et al., 2004). However, CD6 domain 1 mAbs are inhibitory in cellular assays (Kofler et al., 2011; Nair et al., 2010; Singer et al., 1996; Zimmerman et al., 2006), and one has been recently licensed in India for use as an immunosuppressant for psoriasis (Jayaraman, 2013). Inhibition by a CD6 domain 1 mAb depends on the presence of CD6 domain 3, supporting the hypothesis that CD6 domain 1 mAbs perturb CD166/CD6 interactions between cells by steric hindrance (Bowen et al., 1995; Kofler et al., 2011).

Consecutive domains are a feature of group B members of the SRCR domain superfamily (reviewed in Herzig et al., 2010) with three consecutive group B SRCR domains found in the closely related cell surface proteins CD5 and CD6, and the soluble Apoptosis Inhibitor of Macrophages (AIM), also known by other names (Spα, CD5L), which comprises solely three consecutive SRCR domains. The involvement of multiple consecutive SRCR domains in ligand binding has been well characterized for the leukocyte surface receptor CD163, the second and third SRCR domains cooperating in ligand binding to hemoglobin/haptoglobin complexes (Nielsen et al., 2013). All three SRCR domains contribute to ligand binding by AIM (Spα, CD5L), which was found to be associated with immunoglobulin M (IgM) in serum (Arai et al., 2013; Tissot et al., 2002).

The crystal structure of CD6 is the first of consecutive SRCR domains, earlier structures being of single SRCR domains (Garza-Garcia et al., 2008; Hohenester et al., 1999; Rodamilans et al., 2007). We reveal a nonlinear arrangement of the CD6 SRCR domains, which leads us to hypothesize about the roles of the individual domains and their different interactions.

## Results

### Purification, Characterization, and Crystallization of CD6 and CD166

Recombinant His-tagged proteins of human CD6 SRCR domains 1–3 and human CD166 IgSF domains 1–3, i.e. the two N-terminal V domains and one C domain (CD166 VVC), were stably expressed in Lec 3.2.8.1 Chinese hamster ovary (CHO) cells (Chen and Stanley, 2003), purified, and deglycosylated. Protein crystals of the individual proteins were grown and diffraction data collected. We confirmed that the proteins used in this study interact as expected (data not shown and see Supplemental Information) (Bowen et al., 2000; Hassan et al., 2004).

### X-Ray Crystal Structure of CD6 Reveals a Nonlinear Organization of Consecutive SRCR Domains

**Figure 2:**
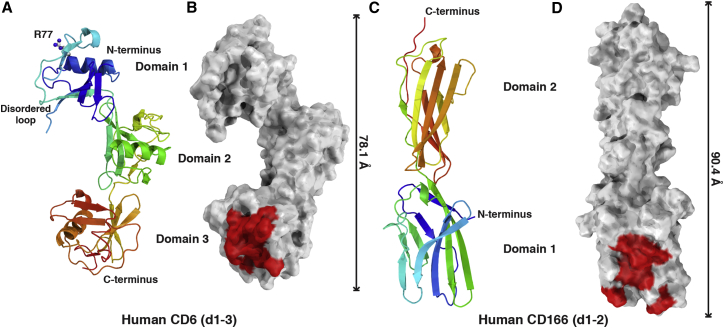
Structures of CD6 and CD166 Identify a Binding Interface and Reveal a Nonlinear Arrangement of Domains in CD6 (A and C) Cartoon diagrams colored from blue at the N terminus to red at the C terminus. (B and D) Surface representations, with mutations in CD6 affecting ligand binding colored red. Similar interdomain orientations of CD6 domains result in an overall nonlinear organization. A disordered loop exists in the N-terminal domain of CD6. Binding of CD6 domain 1 mAb MT605 was specifically reduced by R77A mutation (see Figure S4).

X-Ray diffraction data were collected to 3.15 Å and the structure solved by molecular replacement using a search model derived from the structure of a single SRCR domain of Mac-2 binding protein (PDB: 1BY2, 26.7% sequence identity to CD6 domain 1, 44.5% to CD6 domain 2, and 26.1% to CD6 domain 3; Hohenester et al., 1999) (Table 1). The X-ray structure of CD6 is the first structure for a protein containing consecutive SRCR domains (Figures 2A and 2B).

The structure reveals a nonlinear organization of consecutive SRCR domains of CD6. Despite the interdomain interactions not being conserved, the orientations of each domain relative to its adjacent domain are very similar, both burying approximately 270 Å^2^. A consequence of this orientation at the interfaces between individual SRCR domains is a nonlinear consecutive domain structure, resulting in the three domains spanning 78.1 Å, a shorter distance than if the domains were in a linear arrangement (Figures 2A and 2B). This structural feature was also observed in a lower resolution dataset collected from a different crystal form (data not shown), supporting the idea that this is a biologically relevant structural feature. There is a disordered region in CD6 domain 1 in a similar position to a flexible loop in CD5 domain 1 (Garza-Garcia et al., 2008).

### Mapping Domain 3 Mutants on the CD6 Structure Identifies the Ligand Binding Site for CD166

Previous studies identified CD6 domain 3 and CD166 domain 1 as being sufficient to mediate ligand binding (Bowen et al., 1996; Whitney et al., 1995). The role of the other two CD6 SRCR domains in the heterophilic interaction is not clear. We quantified binding of soluble recombinant CD166 VVC to different forms of streptavidin-immobilized chimeric CD6 by surface plasmon resonance (SPR), in an attempt to detect any contribution of CD6 domains 1 and 2. No difference was detected in the binding of CD166 VVC to CD6, CD6 lacking domain 1, or CD6 lacking domains 1 and 2 (Figure S1; Table S1).

**Figure 3:**
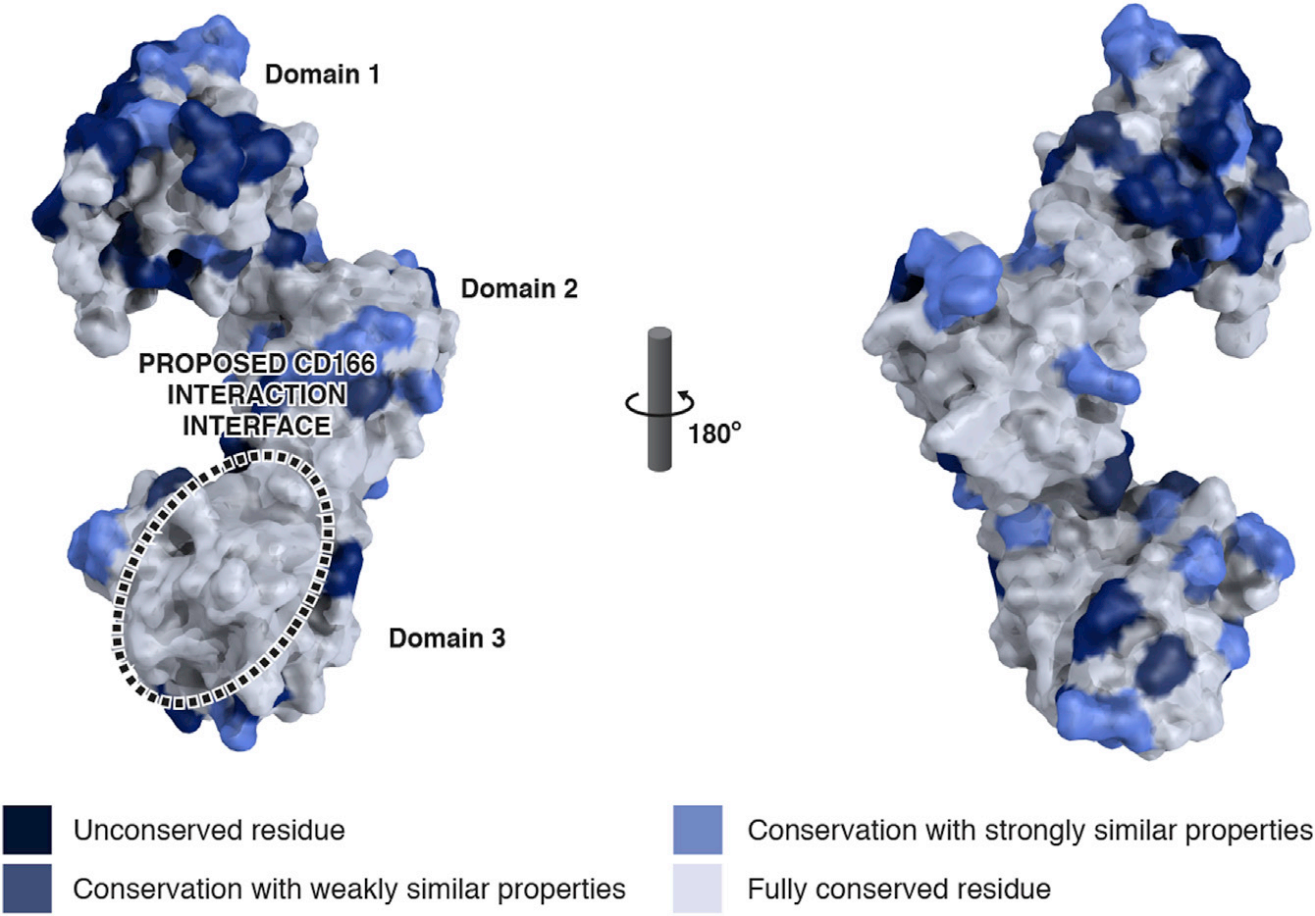
CD6 SRCR Domains, Residues 43–364, which Bind CD166 Are Conserved Across Species Human (UniProt: P30203), mouse (UniProt: Q61003), and rat (UniProt: Q5FVU4) CD6 bind human CD166 (Hassan et al., 2006 and data not shown). Compared with CD6 SRCR domain 3, which binds CD166, CD6 domains 1 and 2 have more variation in amino acid sequence between human, mouse, and rat CD6.

**Figure 4:**
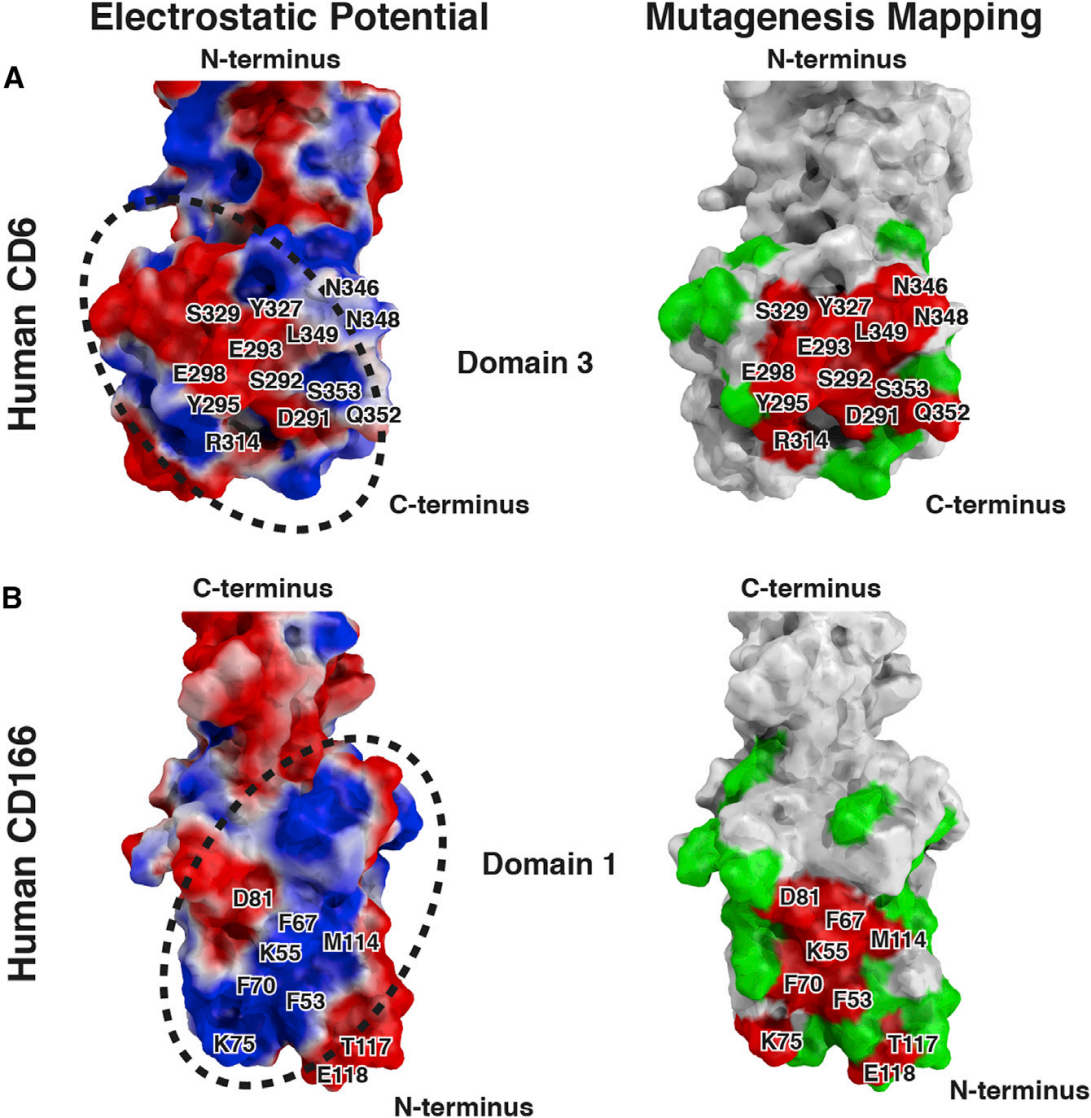
Complementary Electrostatic Potential between CD166 and CD6 Binding Faces Surface representations of human CD6 (A) and human CD166 (B) displaying electrostatic potential (negative charge in red and positive charge in blue) and mutations that do not affect binding (green) and that affect binding (red). Areas of complementary electrostatic potential are outlined with a dotted line.

Using our structure of CD6 and previous mutagenesis data (Bodian et al., 1997; Skonier et al., 1997), we carried out a more extensive mutagenesis study on CD6 domain 3 (Figures 2B and S2; Table S2; see Figure 4A) to define the residues critical for CD166 binding. These data were mapped onto the structure, clearly identifying and defining the boundaries of the surface of CD6 that interacts with CD166. There is a high degree of amino acid conservation in this binding face between human, mouse, and rat CD6 sequences (Figure 3). Within this region amino acids have been identified that reduce binding to CD166, so it is likely that different species use the same face (Bowen et al., 2000) (Figure 3). We extended our previously published cross-species binding analysis to include rat CD6, and showed that human CD166 bound human, mouse, and rat CD6 with the same affinity (Hassan et al., 2006 and data not shown).

### Mapping Domain 1 Mutants on the X-Ray Crystal Structure of CD166 Identifies the Ligand Binding Site for CD6

X-Ray diffraction data for crystals containing CD166 VVC were collected to 1.86 Å and the structure solved by molecular replacement with a search model derived from the second IgSF domain of RAGE (PDB: 3CJJ, 16.4% sequence identity to domain 1 and 30.3% to domain 2; Koch et al., 2010). Only the first two domains could be resolved in the electron density map (Figures 2C and 2D). The crystal lattice did not contain space to accommodate the third domain, suggesting it had been proteolytically cleaved prior to crystal growth.

We mapped previous mutagenesis data that defined residues in CD166 domain 1 critical for CD6 binding (Skonier et al., 1996a, 1996b) onto the crystal structure (Figures 2D; Table S3; see Figure 4B). This confirmed the prediction that binding to CD6 is mediated by the AGFCC′C″ face, in common with many other IgSF interactions (Skonier et al., 1996a, 1996b). We repeated mutagenesis of five of these residues in CD166 domain 1 and confirmed that they all disrupted CD6/CD166 interactions (Figure S3; Table S3).

### There Is Complementary Electrostatic Potential between CD166 and CD6 Binding Sites

Mutagenesis of CD6 domain 3 (Bodian et al., 1997; Skonier et al., 1997) (Table S2 and Figure 4A: N346K, N348R, Q352R) showed that altering the charge from negative to positive in CD6 inhibited CD166 binding. Conversely, altering the charge from positive to negative in CD166 domain 1 (Table S3 and Figure 4B: K55E) inhibited binding to CD6 (Skonier et al., 1996a, 1996b). Loss of charged amino acids (Table S3 and Figure 4B: E118A, K75A, D81A) in CD166 domain 1 also reduced binding, consistent with electrostatic potential being important for ligand binding. The calculated electrostatic potentials of the proposed interface between CD6 and CD166 are compatible with their interaction. The CD6 surface features a stripe of negative charge flanked with positive charge, and the CD166 surface features a complementary positively charged stripe flanked by negative charge (Figure 4).

### Mapping Domain 1 mAb Epitopes on the CD6 Structure Shows How Ligand Binding between Cells Might Be Disrupted

Inhibition by CD6 domain 1 mAbs has only been observed between cells, suggesting that intermembrane dimensions are important for their effects (Bowen et al., 1996, 1995). To gain some insight from the CD6 structure as to how CD6 domain 1 mAbs might disrupt CD6/CD166 interactions, we mapped the epitope of a CD6 domain 1 mAb, MT605, which has been shown to inhibit immune responses between cells expressing CD6 and CD166 (Kofler et al., 2011; Singer et al., 1996; Zimmerman et al., 2006). To identify the epitope, we produced alanine mutants of a number of surface-exposed residues in CD6 domain 1. Binding of MT605 was abolished by R77A (Figure S4). Binding of another CD6 domain 1 mAb, MEM-98, was unaffected by R77A but was eliminated by E63A. MT605 was unaffected by E63A (Figure S4). In the orientation shown in Figure 2, R77 lies at the top of CD6 domain 1. A large antibody binding in this position may prevent the close approach between cell membranes needed for CD6/CD166 engagement.

### CD6 Nonsynonymous SNPs Associated with Multiple Sclerosis which Alter Expression Are Buried

**Figure 5:**
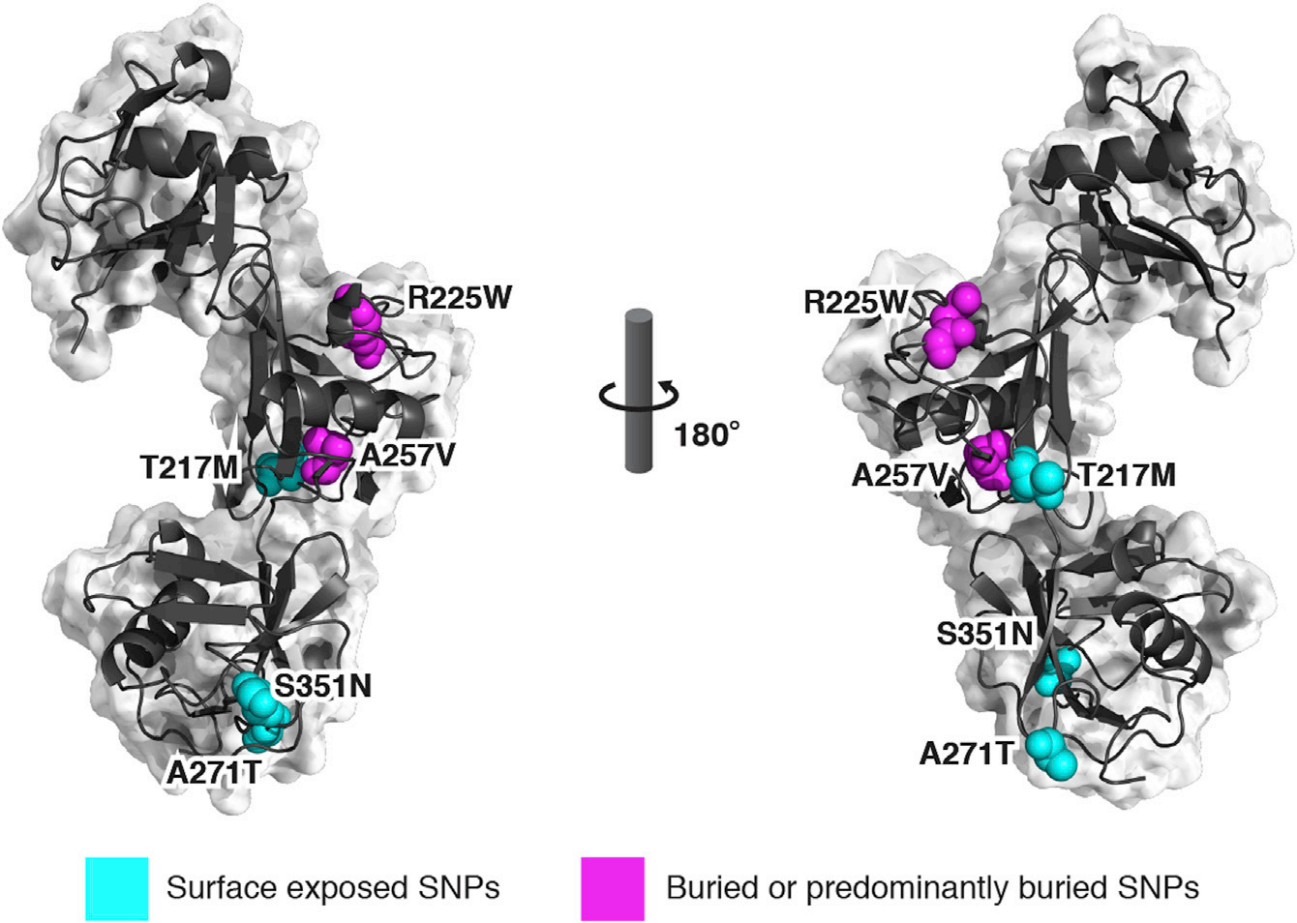
Nonsynonymous SNPs in CD6 Associated with Disease Are Not in the CD166 Binding Site SNPs are mapped on the structure of CD6 and displayed as spheres.

Of the SNPs identified in CD6, five alter amino acids in domains 2 and 3 (Figure 5). Homozygotes for two of these SNPs (R225W and A257V) in domain 2 have been associated with susceptibility to multiple sclerosis (Swaminathan et al., 2013). These two residues are buried within the domain and may disrupt the domain structure and stability of the molecule, explaining the correlation between reduced expression and susceptibility to multiple sclerosis.

### The CD6 SNP, S351N, Results in Glycosylation that Disrupts CD6/CD166 Interactions

Of the surface-exposed SNPs in CD6, the change from S351 to S351N found in 10% of the genomes analyzed (http://www.1000genomes.org) is the only residue close to the ligand binding site. We mutated CD6 S351 to S351N and compared the binding of soluble CD166 VVC to the two variants by SPR. CD166 VVC bound with a 10-fold weaker affinity to CD6 S351N compared with CD6 S351 (Figure S5; Table S5). Analysis of the kinetics of binding showed an increase in the dissociation rate of CD6 S351N compared with CD6 S351 (Figure S5; Table S4).

As S351N introduces a consensus N-linked glycosylation site (NQS), binding of CD166 may be sterically hindered by carbohydrate. There are seven consensus N-glycosylation sites in the three extracellular domains of CD6. In the recombinant protein, we observed electron density for GlcNAc attached to residue N229 in the second domain. There was no observable electron density for the other potential glycosylation sites in CD6. Mass spectrometry was used to determine whether recombinant CD6 S351N is glycosylated. As glycosylated peptides are not identified by liquid chromatography-tandem mass spectrometry due to the unknown mass of the N-linked carbohydrate, we quantified the precursor ion intensity obtained from extracted ion chromatograms of the FNNSNLCS_351_QSLAAR and FNNSNLCN_351_QSLAAR tryptic peptides before and after deglycosylation (Figure S6; Table S5). The S351 peptide was not glycosylated (Figures S6A and S6B), whereas the ion intensity of the S351N peptide was dependent on deglycosylation, showing that it was glycosylated (Figures S6C and S6D). Consistent with the interpretation that S351N disrupted ligand binding by introducing a bulky carbohydrate moiety in proximity to the CD166 binding site, the CD6 mutant S351A restored binding, as measured by SPR (Figure S5; Table S5).

The glycosylation states of the other putative glycosylation sites in CD6 were determined qualitatively from the mass spectrometry data by comparing the number of peptide spectral matches of peptides containing each N-linked glycosylation site before and after deglycosylation with peptide-*N*-glycosidase F, showing that N28, N49, N229, and N351 are glycosylated and that N339 appears to be partially glycosylated (Table S5).

### Heterodimers Are Formed at the Expense of CD6 and CD166 Homodimers

**Figure 6:**
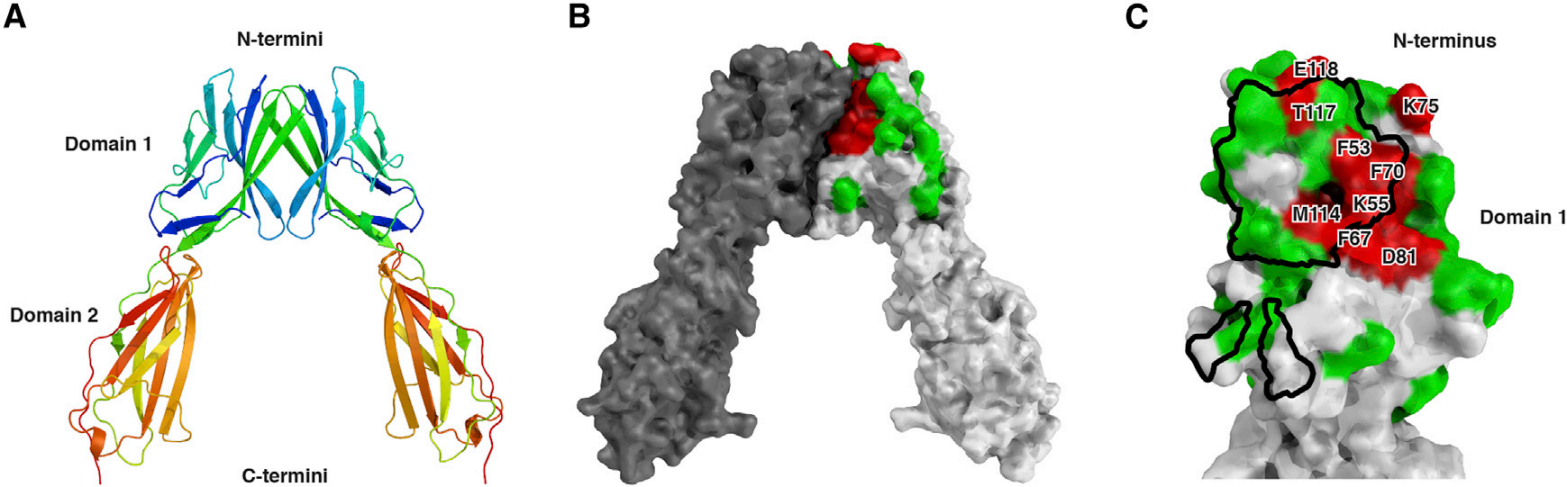
Crystal Contacts Identify a Potential CD166 Domain 1 Homophilic Binding Face Cartoon representations colored from blue at the N-terminus to red at the C-terminus (A) and surface representations (B) of CD166 domains 1 and 2, showing a binding interface between the A′GFCC′C″ faces (Bowen et al., 2000). The homophilic interface is outlined in black (C).

Homophilic and heterophilic interactions of CD166 are mediated by the N-terminal domain. Mutations in the A′GFCC′C″ face, which disrupt CD6/CD166 interactions (Figure 2), map to the crystal contacts between the two N-terminal domains in the CD166 structure (Figure 6) (Bowen et al., 2000).

**Figure 7:**
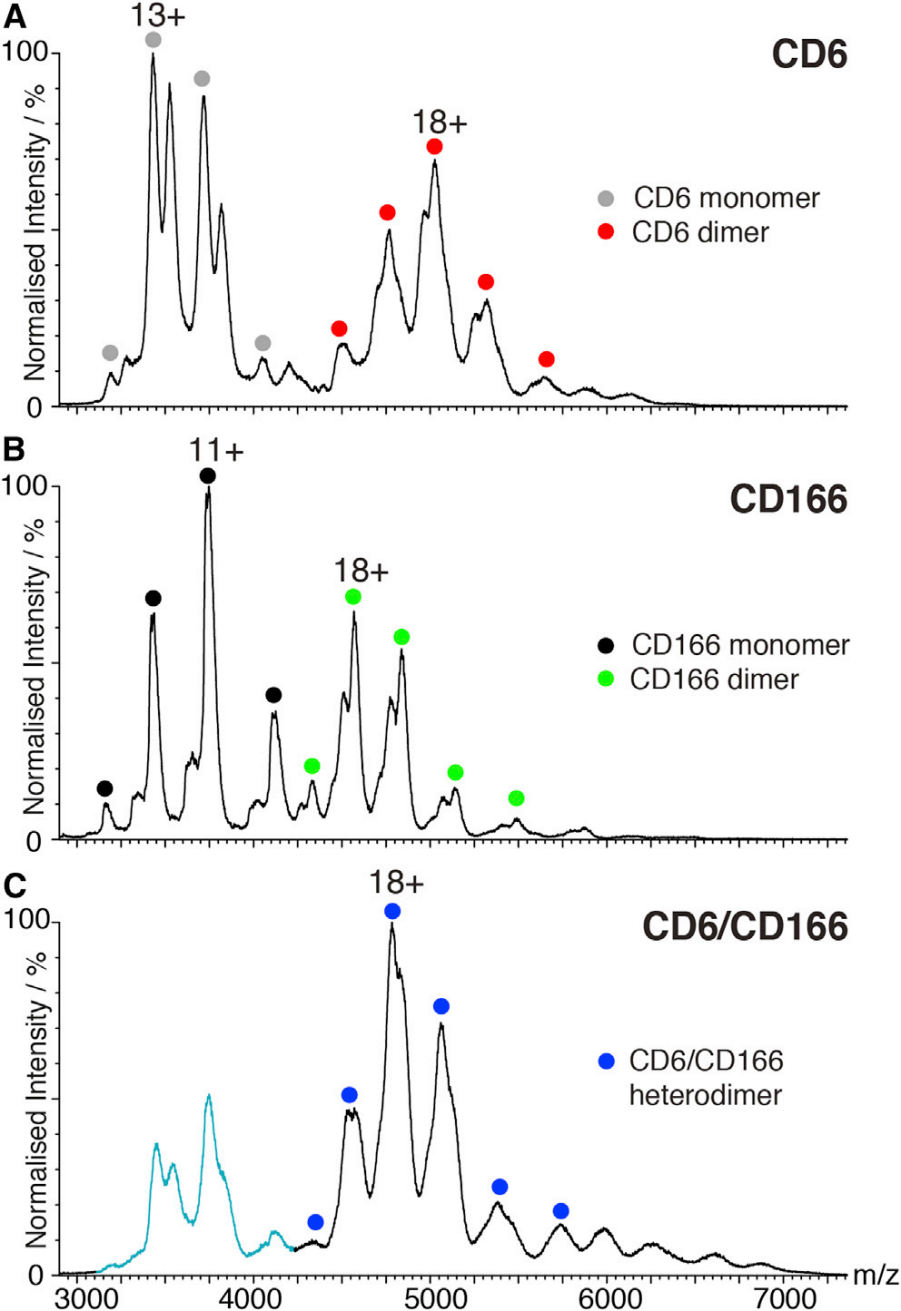
Heterodimers Are Formed at the Expense of CD6 and CD166 Homodimers Native mass spectrometry data are shown for the CD6 (A) and CD166 (B) proteins separately, and for the CD6/CD166 mixture (C). The CD6 and CD166 proteins exist predominantly in a monomer-dimer equilibrium. Charge state series are indicated with circles: gray and red for CD6 monomer and dimer; and black and green for CD166 monomer and dimer, respectively. In the mixture (C), the CD6/CD166 heterodimer, indicated with blue circles, is the most abundant species. Smaller amounts of individual CD6 and CD166 monomers, colored in cyan, also exist. Where more than one mass was found for a species due to glycosylation, only the most abundant one is indicated on the spectra for clarity. All experimentally calculated masses can be found in Table 2.

If the heterophilic and homophilic binding sites overlap, as implied by the CD166 crystal contacts, homophilic CD166 interactions would be disrupted by the formation of heterodimers with CD6. We examined the molecular species present when CD6 and CD166 were mixed in comparison with the individual components by native mass spectrometry. When CD6 and CD166 were analyzed separately, we found that both CD6 and CD166 were predominantly in monomer-dimer equilibrium (Figures 7A and 7B). When CD6 and CD166 were mixed, there was an increase in the relative abundance of heterodimers, a corresponding decrease of the CD6 and CD166 monomers, and a complete depletion of homodimers when compared with the unmixed samples (Figure 7C). These data show that heterodimers are formed at the expense of CD6 and CD166 homodimers. Due to the heterogeneity in molecular masses caused by glycosylation, the collision voltage on the mass spectrometer was increased to achieve more accurate mass determination, and these experimentally calculated masses are shown in Table 2. The mass of the heterodimer was confirmed by analysis of its composition by tandem mass spectrometry (data not shown).

Comparison of the molecular species identified in native mass spectrometry and multi-angled light scattering also revealed a dynamic equilibrium between the different species (Figure S7).

## Discussion

The structures of CD6 and CD166 give insight into the heterophilic CD6/CD166 and homophilic CD166/CD166 interactions and those of other proteins containing consecutive SRCR domains. The structure of CD6 is the first for proteins containing consecutive SRCR domains. One striking feature is the nonlinear domain organization, which has implications for the topology and orientations of interactions with individual domains.

### The Role of Consecutive SRCR Domains in CD6/CD166 Interactions

The relatively high affinity *trans* interaction between CD6 and CD166 on apposing cells is mediated by the CD6 membrane proximal domain. The main contribution of the other two CD6 SRCR domains to the CD6/CD166 interaction may be to regulate accessibility of CD166 to the membrane proximal domain of CD6, as formation of CD6/CD166 heterodimers involves competition with the homophilic CD166 interactions. *Trans* CD166 homophilic interactions were inhibited by preventing clustering in *cis* on the cell surface with an antibody specific for the membrane proximal domains of CD166 or by a dominant negative mutant lacking the N-terminal domain (van Kempen et al., 2001, 2004; Weidle et al., 2010). Weak interactions in *cis* between the membrane proximal domains of CD166 at the cell surface may enhance homophilic adhesion in *trans* (van Kempen et al., 2001; Wu et al., 2011). The projection of CD6 domain 1, which results from the nonlinear domain organization, may be important for preventing clustering of CD166 at the cell surface, and may provide an additional steric hindrance mechanism to disrupt the CD166 *trans* homodimers between cells.

The topology of the CD6 SRCR domains shows how CD6 domain 1 mAbs might impede access of CD166 to the membrane proximal domain of CD6 between cells (Bowen et al., 1995; Kofler et al., 2011). Inhibition of the CD6/CD166 interaction by CD6 domain 1 mAbs has only been observed between cells and is likely to be dependent on the level of CD6/CD166 engagement (Bowen et al., 1995; Castro et al., 2007; Kofler et al., 2011; Singer et al., 1996; Zimmerman et al., 2006).

The main role of domain 2 of CD6 may be as a spacer to maintain the projection of CD6 domain 1. However, in the characterization of an interaction between the SRCR domains of the closely related protein CD5 and the V region of the heavy chain of immunoglobulin, first reported in rabbit, only the isolated domain 2 of human CD5 was capable of binding the V region of the heavy chain of human immunoglobulin (Pospisil et al., 2000). CD5 differs in topology from CD6 in containing a proline-rich linker between domains 1 and 2 that may be important for accessibility to domain 2.

A subsidiary role for domains other than CD6 domain 3 and CD166 domain 1 in CD6/CD166 interactions, which are below the detection limit of SPR analysis, cannot be completely ruled out, as there are data suggesting weak interactions of other domains in high-avidity binding assays (Bowen et al., 1996). In the context of a lattice formation between cells (Weidle et al., 2010), a very low-affinity interaction may be productive (Wu et al., 2011). CD6 domain 2 may be important for divalent cation-dependent interactions of CD6, as it contains a conserved triacidic motif D187/D188/E253 (Nielsen et al., 2013). CD6/CD166 interactions occur in the absence of divalent cations (Hassan et al., 2004; Patel et al., 1995; L.I.G., unpublished data).

Homodimers of CD166 and also CD6 were detected in native mass spectrometry (Figure 7). When mixed, the main species was heterodimers of CD6 and CD166. Based on data for CD5, CD6 homodimers are more likely to form in *cis* on the same cell surface (Bamberger et al., 2011; Brown and Lacey, 2010). The nonlinear arrangement of the SRCR domains in CD6 is compatible with individual domains engaging in interactions in different orientations. If the angle of projection of CD6 from the cell surface is as depicted in Figures 1 and 2, it places the N-terminal domain in a position that could be important for creating contacts on the same cell surface, including homophilic interactions. Deletion of the N-terminal domain of CD6 resulted in a protein that was not stable at the cell surface, whereas CD6 containing domain 1 but lacking the membrane proximal domain is expressed stably at the cell surface (Castro et al., 2007; Kofler et al., 2011; M.H.B., unpublished data). Dimerization of CD6 might be important for stabilization at the surface in the absence of ligand binding, and ligand engagement by CD166 may be important for stabilizing CD6 monomers at the cell surface to maintain immune responses (Zimmerman et al., 2006).

### Heterophilic CD6/CD166 and Homophilic CD166/CD166 Interactions

In the competition between CD6 domain 3 and the N-terminal domain of CD166, heterodimers are formed at the expense of the weaker homophilic interactions of CD166 (Hassan et al., 2004; Te Riet et al., 2007; and see above). Crystal contacts in the CD166 structure suggest that the sites for heterophilic and homophilic binding on CD166 domain 1 overlap (Figure 6). Alternatively, heterophilic and homophilic interactions may compete through steric hindrance.

The structures of the interacting domains, the membrane proximal domain of CD6, domain 3, and the N-terminal domain of CD166 showed that mutants that disrupt binding to CD166 cluster together on the surface of CD6 (Bodian et al., 1997; Skonier et al., 1997), identifying the binding face on domain 3. Complementary mapping of mutants of CD166 that affected ligand binding delineated the binding site predicted to be on the A′GFCC′C″ face of the CD166 structure (Bodian et al., 1997; Skonier et al., 1997).

The binding faces of CD6 SRCR domain 3 and CD166 domain 1 are predominantly positively and negatively charged, respectively (Figure 4) (Bowen et al., 2000). Ligand binding by SRCR domains in MARCO and CD163, in groups A and B, respectively, involves complementarity in charge (Nielsen et al., 2013; Ojala et al., 2007). Electrostatic potential is likely to be important in the CD6/CD166 interaction.

### Implications for Other Proteins Containing Consecutive SRCR Domains

The interaction between CD6 and CD166 functions in the adaptive immune system, whereas the majority of SRCR superfamily domains are found in the innate immune system (Herzig et al., 2010). Preservation of the three consecutive SRCR domains may be important for maintaining more promiscuous low-affinity interactions (Abdi et al., 2014; Arai et al., 2013) with ligand binding depending on avidity with contributions from each domain, as has been observed in the interaction between AIM (Spα, CD5L) and IgM (Arai et al., 2013). There may be a similar nonlinear domain arrangement in other proteins containing consecutive SRCR domains that is important for multiple domains making contact with a ligand. Modeling the conformation of longer arrays of SRCR domains with similar length linker sequences suggests that these proteins would form a helical structure.

Group A SRCR domains containing proteins that consist of polypeptides with single SRCR domains trimerize, which may create an alternative topology for multivalent binding. There is a model based on a crystal structure for how membrane distal single SRCR domains of MARCO multimerize to form a large ligand binding face for pathogens (Ojala et al., 2007). Direct pathogen binding has been described for several SRCR domains, including CD6 (Sarrias et al., 2007). A bacterial binding peptide motif was first identified in an SRCR domain of DMBT1, otherwise known as salivary agglutinin (Bikker et al., 2004; Madsen et al., 2010). A consensus sequence for this bacterial binding peptide is found in CD6 SRCR domain 2 (Bikker et al., 2004). Examining the position of this peptide on the structure of CD6, the region is predominantly surface exposed and is on the opposite face to the CD166 binding site. Its significance is uncertain. Mutation of this consensus sequence in the SRCR domain protein WC1 did not affect bacterial binding (Hsu et al., 2015).

### Effects of Single Polymorphisms in CD6

Selection pressure for nonsynonymous SNPs in CD6 domain 2 that are associated with susceptibility to multiple sclerosis (Figure 5) may simply be to reduce expression levels (Swaminathan et al., 2013). Reduced expression levels will lower both the threshold of activation and ligand engagement (Hassan et al., 2006). The strongest association of a SNP in CD6 with disease susceptibility is in an intron, and correlates with a reduced ratio of expression of full-length CD6 compared with a form of CD6 which lacks the ligand binding domain, suggesting that the dominant effect is mediated by decreased ligand binding (Castro et al., 2007; Kofler et al., 2011). Disease association for the less common SNP, S351N, which has reduced affinity for CD166, has not yet been reported. Mutagenesis and mass spectrometric analysis provided evidence that reduced binding was due to glycosylation of CD6 at S351N. We assume this high frequency SNP is likely to be associated with subtle functional changes in CD6 activity.

### Topology of CD6/CD166 Interactions

On engagement by CD166, CD6 has been observed to move into areas of close contact between T cells and antigen-presenting cells (Castro et al., 2007; Gimferrer et al., 2004; Zimmerman et al., 2006), suggesting that the interacting receptors will match the dimensions of the T-cell receptor and other receptors observed to colocalize, ∼140 Å (Dushek et al., 2012) (Figure 2). The nonlinear domain structure may be important for accommodating CD6 in areas of close apposition between cells. The nonlinear structure of the three SRCR domains spans 78.1 Å. This excludes the 37 amino acid membrane proximal stalk region of unknown dimensions. The two IgSF V domains of CD166 span 90.4 Å, comparable with the dimensions of the two IgSF V domains in another cell surface receptor, JAM (86 Å, PDB: 1F97). Extrapolating from the dimensions of the two CD166 IgSF V and three C domains taking the size of a C domain to be 35 Å from human CD2 domain 2 (PDB: 1HNG), the five domains in a linear array would be predicted to span ∼200 Å. This suggests there is flexibility and/or deviation from the perpendicular by CD166 between apposing cells to optimize formation of *trans* interactions, as has been proposed for five domain E-cadherins (Wu et al., 2011). If the parallel, as distinct from anti-parallel orientation of the interacting CD166 N-terminal domains, is physiological, it will restrict the dimensions of homophilic *trans* interactions (Te Riet et al., 2014; van Kempen et al., 2001). Determination of the X-ray crystal structures of CD6 and CD166 allows us to create a more accurate model of how these proteins regulate interactions at the cell surface, and to design the most effective therapeutic reagents.

## Experimental Procedures

### Recombinant Proteins for Crystallization

Three human CD6 (GenBank: U34623; UniProt: P30203) SRCR domains (residues 1–364) and two V-like domains and one C-like domain (VVC) (residues 1–335) of human CD166 (UniProt: Q13740) were amplified from plasmid DNA (Bowen et al., 1996) and expressed as endoglycosidase H sensitive His-tagged proteins (CD6; LCSASRGHHHHHH CD166; YLDLSTRHHHHHH) containing their native leader sequences using PEE14 vector in CHO Lec 3.2.8.1 cells (Chen and Stanley, 2003). CHO cell lines selected for stable expression were grown in cell factories to confluence; sodium butyrate (2 mM) was then added and the cells were allowed to secrete for 2–3 weeks before harvesting. Tissue culture supernatant was concentrated (Sartorius Vivablock, molecular weight cutoff 10 kDa) and proteins purified using nickel chromatography columns (NiNTA, Qiagen) and elution with an imidazole gradient in 10 mM HEPES, 150 mM NaCl (pH 7.4). Proteins were immediately dialyzed to remove imidazole and then subjected to size-exclusion chromatography (Superdex200 column; GE Healthcare). For crystallization, proteins were deglycosylated with EndoHf (New England Biolabs), 0.3–2.5 U/ml in 10 mM HEPES, 150 mM NaCl (pH 7.4) at 37°C for 2 hr and analyzed by SDS-PAGE.

### Crystallization

The three extracellular SRCR domains of human CD6 were crystallized using the sitting drop method in 0.1 M ammonium sulfate, 0.3 M sodium formate, 0.1 M sodium cacodylate, 3% w/v PGA-LM, 20% MPD (pH 6.5) at 20°C. Drops were set up using 2.5 μl of a protein solution containing a complex of CD6 and CD166 at an OD_280_ of 2.22, and 2.5 μl of the reservoir solution. CD6 crystals grew within 4 weeks.

The two membrane distal immunoglobulin domains of human CD166 were crystallized using the sitting drop method in 0.1 M sodium HEPES (pH 7.5) and 25% w/v PEG-2000 MME. Drops were set up using 2 μl of a protein solution containing CD166 domains 1–3 at an OD_280_ of 4.58. Crystals grew after 4 weeks. Crystals were flash-frozen in liquid nitrogen and data were collected at the Diamond Light Source, Harwell, at 100 K on beamline i04-1.

### Data Collection and Processing

Native datasets were collected for CD6 and CD166, to 3.15 Å and 1.86 Å, respectively. The data were integrated and scaled using XDS (Kabsch, 2010) and aimless (Evans and Murshudov, 2013) as implemented in XIA2 (Winter et al., 2013). Molecular replacement search models were generated using CHAINSAW (Stein, 2008), and large loops removed. The structure of human CD6 was solved by searching for three copies of a search model derived from the Mac2 binding protein, a single SRCR domain (PDB: 1BY2; Hohenester et al., 1999) using PHASER (McCoy et al., 2007), part of the CCP4 software suite (Winn et al., 2011). The human CD166 structure was solved by searching with a search model derived from the second immunoglobulin domain in the ligand binding domain of human RAGE (PDB: 3CJJ; Koch et al., 2010). Model building and refinement were carried out using Coot (Emsley et al., 2010) and AUTOBUSTER (Bricogne et al., 2011). The CD6 and CD166 structures were refined to *R*_work_/*R*_free_ of 0.25/0.28 and 0.22/0.24, respectively. Coordinates were deposited in the PDB (PDB: 5a2e for CD6 and PDB: 5a2f for CD166).

### Native Mass Spectrometry

Purified CD6 and CD166, along with a mixture of both proteins, were subjected to native mass spectrometry analysis. Proteins were at a concentration of 20 μM and the mixture formed by incubating an equal amount of CD6 and CD166 (both at 20 μM) for 30 min at room temperature. Samples were buffer exchanged from 10 mM HEPES, 150 mM NaCl (pH 7.4) into 250 mM ammonium acetate (pH 7.6) using Biospin-6 columns (Bio-Rad). Mass spectrometry measurements were carried out on a modified Synapt G1 HDMS (Waters Corp.) Q-ToF mass spectrometer (Bush et al., 2010). The instrument was mass calibrated using a solution of 10 mg/ml cesium iodide in water. Aliquots (2 μl) of samples were delivered to the mass spectrometer by means of nano-electrospray ionization via gold-coated capillaries, prepared in-house (Hernandez and Robinson, 2007). Instrumental parameters were as follows: source pressure 5.6 mbar, capillary voltage 1.20 kV, cone voltage 50 V, trap energy 10 V, bias voltage 5 V, and trap pressure 1.63 × 10^−2^ mbar. The trap energy was increased to 30 V to obtain more accurate mass measurements.

## Author Contributions

P.E.C. purified and crystallized protein, solved the human CD6 and CD166 X-ray crystal structures, made the figures, and contributed to writing of the manuscript. L.I.G. purified protein and performed SPR experiments. J.Y., C.V.R., and C.M. carried out native and glycosylation mass spectrometry experiments, respectively. D.H. produced cell lines. S.M.L. and S.J. solved the human CD6 and CD166 X-ray crystal structures. M.H.B. directed the project and wrote the manuscript.

## Figures and Tables

**Table 1:** Crystallographic Data for CD6 and CD166

	CD6	CD166
**Data Collection**		

X-Ray source	Diamond Light Source, beamline i04-1	Diamond Light Source, beamline i04-1
Wavelength (Å)	0.92	0.92
Resolution range (Å)	77.93–3.15 (3.23–3.15)	51.14–1.86 (1.91–1.86)
Space group	P 63 2 2	P 43 21 2
Unit Cell		
a, b, c (Å)	161.48, 161.48, 93.85	72.32, 72.32, 105.04
α, β, γ (°)	90, 90, 120	90, 90, 90
Total reflections	69,680 (5,176)	313,66 (22,938)
Unique reflections	12,884 (933)	24,110 (1,731)
Multiplicity	5.4 (5.5)	13.0 (13.3)
Completeness (%)	99.6 (99.8)	99.9 (99.9)
Mean *I*/σ(*I*)	7.2 (2.2)	20.1 (3.8)
*R*_merge_ (all I^+^ and I^−^) (%)	23.2 (80.0)	8.0 (65.9)

**Refinement**		

Resolution range (Å)	77.93–3.15	21.09–1.86
No. of reflections (work/test set)	12,883/629	24,029/1,229
No. of atoms (protein, glycans, ligands, waters)	4,399, 27, 60, 43	3,496, 54, 160, 186
Protein residues	324	218
Mean B-factors (protein, glycans, ligands, waters) (Å^2^)	37.85, 61.24, 35.33, 10.28	32.53, 47.28, 59.05, 38.80
*R*_work_ (%)	25.31	22.17
*R*_free_ (%)	28.31	23.91
Rmsd from ideal values (bonds, Å)	0.0061	0.0061
Rmsd from ideal values (angles, °)	0.76	0.93
Ramachandran plot		
Favored regions (%)	95.05	96.79
Outliers (%)	0	0

Rmsd, root-mean-square deviation.

**Table 2:** Calculated Mass Species of CD6 and CD166 from Native Mass Spectrometry

	Experimentally Calculated Mass (Da)	Standard Deviation (Da)
**CD6**		

Monomer 1	44,509	±3
Monomer 2	45,721	±4
Dimer 1	89,135	±13
Dimer 2	90,173	±16
Dimer 3	91,436	±13

**CD166**		

Monomer 1	39,751	±8
Monomer 2	40,139	±12
Monomer 3	40,975	±4
Monomer 4	41,198	±6
Dimer 1	80,045	±9
Dimer 2	81,046	±24
Dimer 3	82,112	±24

**CD6/CD166**		

Dimer 1	84,557	±40
Dimer 2	85,592	±32
Dimer 3	86,770	±24

## Supplemental Information

Document S1. Figures S1–S7, Tables S1–S5, and Supplemental Methods

Document S2. Article plus Supplemental Information
