# Supplementary material for: Structures of CD6 and Its Ligand CD166 Give Insight into Their Interaction
Source: Structure. 2015 Aug 4;23(8):1426–36. doi: 10.1016/j.str.2015.05.019 (PMC4533223; doi:10.1016/j.str.2015.05.019)
Supplement: Document S1. Figures S1–S7, Tables S1–S5, and Supplemental Methods [file mmc1.pdf]

Structure, Volume *23*

Supplemental Information

Structures of CD6 and Its Ligand CD166 Give Insight into Their Interaction

Paul E. Chappell, Lee I. Garner, Jun Yan, Clive Metcalfe, Deborah Hatherley, Steven Johnson, Carol V. Robinson, Susan M. Lea, and Marion H. Brown

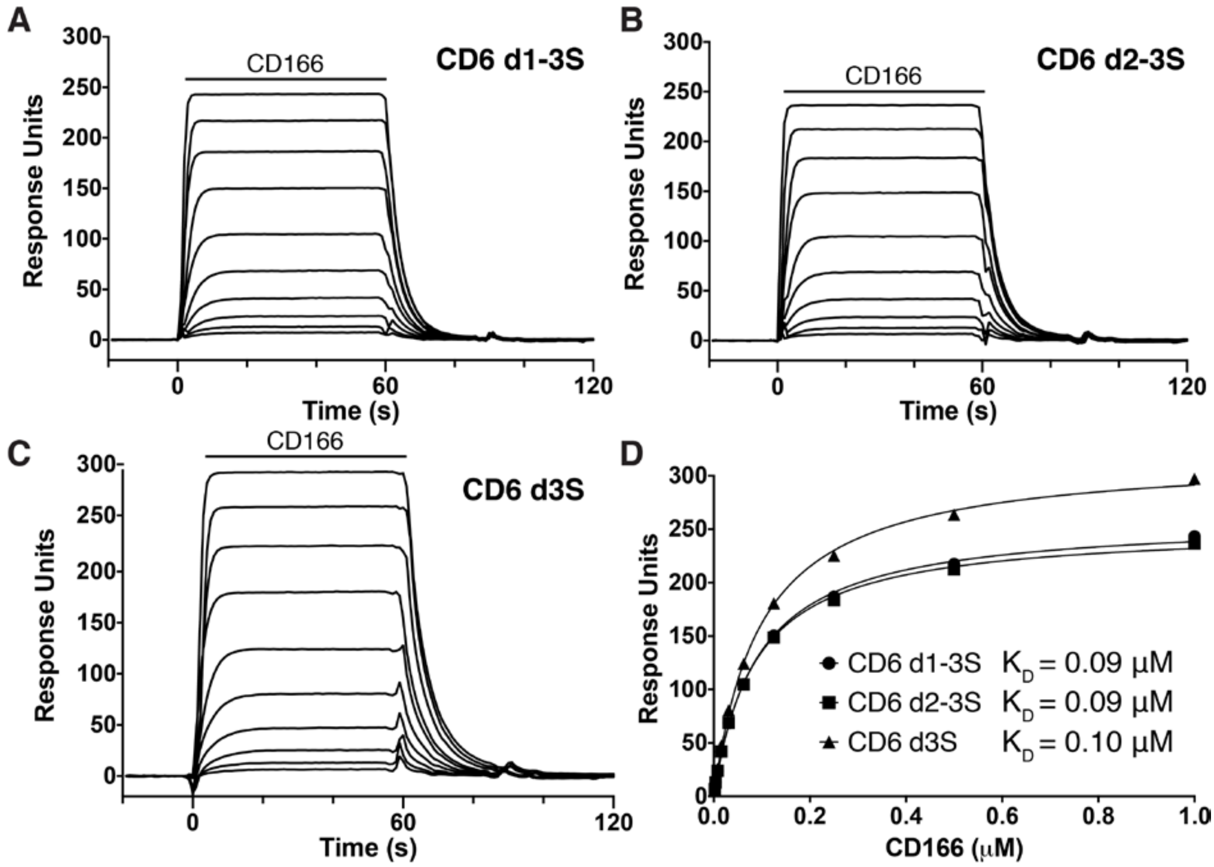

Figure S1 related to Figure 2. There is no detectable contribution of CD6 domains 1 and 2 to the interaction with CD166 as measured by SPR at 37 ºC. Increasing concentrations of soluble CD166 VVC were injected at 37 ºC, over CD6 domains 1-3 (A), CD6 domains 2-3 (B) or CD6 domain 3 (C), plus stalk (S) and rat CD4 d3+4 in a control flow cell immobilised via the biotin tag to streptavidin. (A-C) Sensorgram traces with response over control flow cell subtracted are shown. Bars represent injection period. (D) Specific binding at equilibrium was calculated by subtracting the response over rat CD4 d3+4. The equilibrium dissociation constants, K_D, were calculated by curve fitting (Table S1). Top protein concentration: CD166, 1.0 µM; plus nine two-fold serial dilutions were used for the equilibrium binding experiments.

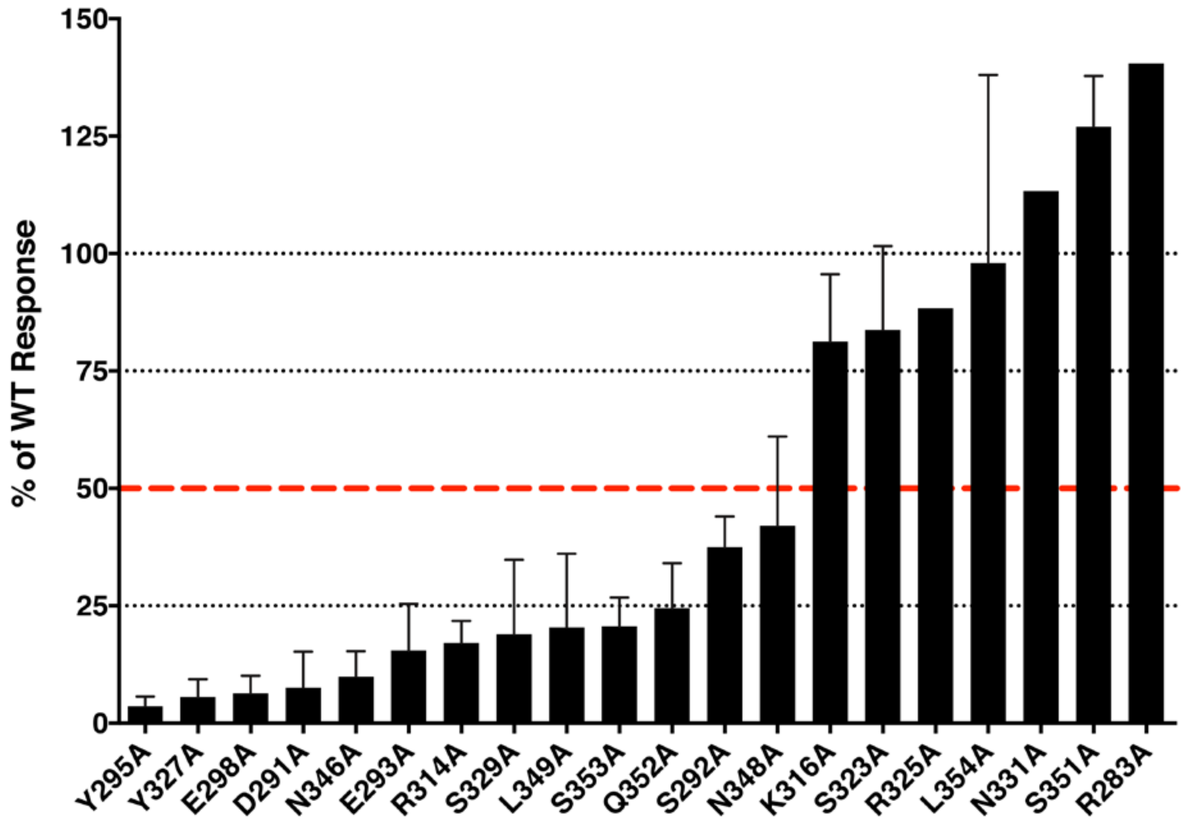

Figure S2, related to Figure 2. Mutation of residues in CD6 domain 3 disrupts CD6/CD166 interactions. Twenty surface exposed residues in CD6 domain 3 were mutated to alanine. Soluble CD166d1-5 at approximately the K_D (0.1 µM) was injected at 25^{0}C, over CD6-CD4d3+4 chimeras immobilised via OX68 mAb. Equilibrium binding data for the mutants are plotted as the mean percentage (±S.E.M) of wild type response normalised to 100 %. The dashed line indicates 50 % response which was used to define residue involvement in binding. There were equivalent levels of all proteins immobilised as monitored by binding of a CD6 domain 3 mAb (OX126).

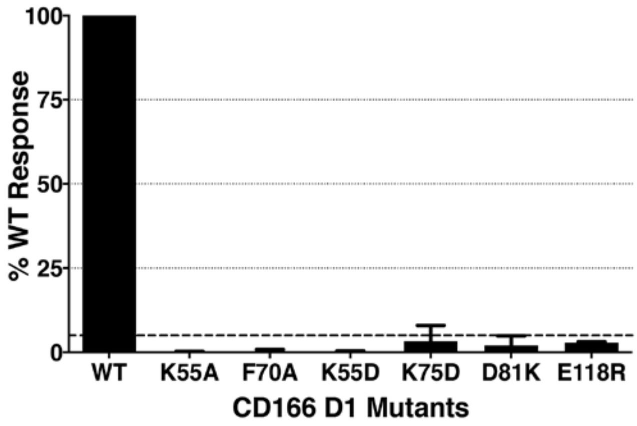

Figure S3, related to Figure 2. Mutation of residues in the CD166/CD166 crystal contacts disrupt CD6/CD166 interactions. Five surface exposed residues in CD166 domain 1 were mutated. Soluble CD6 His at approximately the K_D (0.1μM) was injected at 25^0C, over CD166-CD4d3+4 chimeras immobilised via OX68 antibody. Equilibrium binding data for the mutants are plotted as the mean percentage (±S.E.M) of wild type (WT) response normalised to 100 %. Each mutant reduced binding by ≥ 95% (dashed line). There were equivalent levels of all proteins immobilised as monitored by binding of a CD166 mAb. Expression levels of K55A and K55D were low as previously reported (Skonier et al., 1996a).

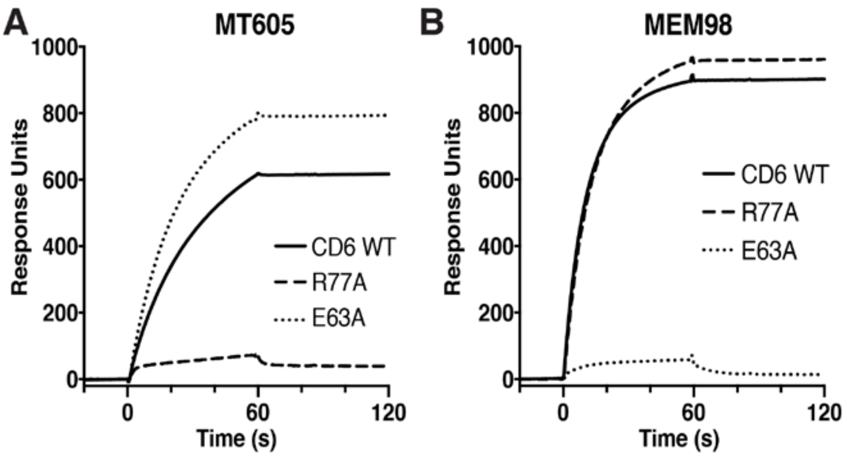

Figure S4, related to Figure 2. A CD6 mAb which inhibits T cell activation binds to the top of CD6 domain 1. In SPR analysis, MT605 (A) and MEM98 (B) were injected at 10 µg/ml at 25 ºC, over CD6d1-3S, CD6d1-3S mutants, R77A and E63A or rat CD4 d3+4 in a control flow cell immobilised via OX68 mAb at. Sensorgram traces with response over control flow cell subtracted are shown. Binding of MT605 was specifically reduced by the R77A mutation and not by E63A. There were equivalent levels of all proteins immobilised as monitored by binding of a CD6 domain 3 mAb (OX126).

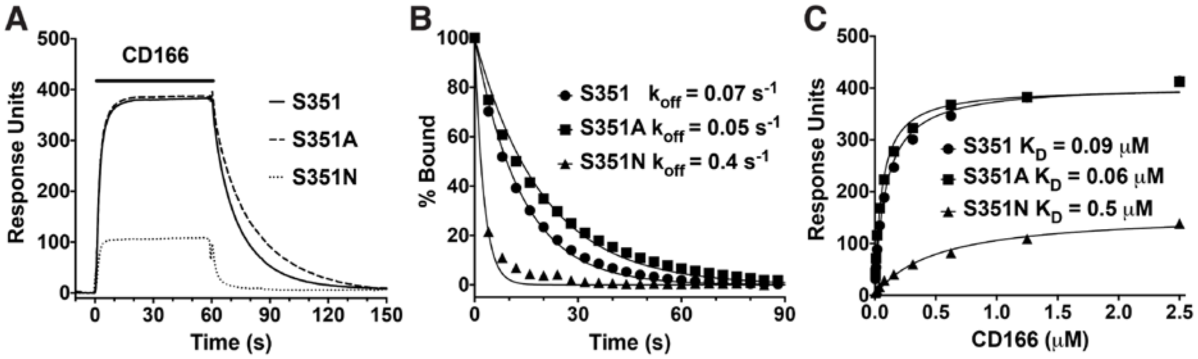

Figure S5, related to Figure 5. A CD6 SNP, S351N in the binding site reduced affinity for CD166. Increasing concentrations of soluble CD166 VVC were injected at 25 °C, over immobilised CD6 S351, CD6 S351A, CD6 S351N and rat CD4 d3+4 in a control flow cell immobilised via OX68 mAb. (A) Sensorgram traces with response over control flow cell subtracted. Bar represents injection period. (B) Dissociation curves for CD166 VVC over immobilised CD6 S351, S351A, or S351N, with response in control flow cell subtracted. The response at t=0 was normalised to 100 % for each flow cell and dissociation monitored over time. The dissociation rate constant, $K_{off}\,s^{-1}$, was calculated for each interaction by non-linear regression using Graphpad Prism (dissociation – one phase exponential decay). (C) Specific binding at equilibrium was calculated by subtracting the response over the control flow cell. The equilibrium dissociation constants, K_D were calculated by curve fitting (Table S4). Top protein concentration: CD166, 2.5 μM; plus nine two-fold serial dilutions were used for the equilibrium binding experiments. There were equivalent levels of all three proteins immobilised as monitored by binding of a CD6 domain 3 mAb (OX126). It is likely that a higher proportion of the immobilised CD6 S351N was inactive leading to a reduction in stoichiometry of binding compared with CD6 S351.

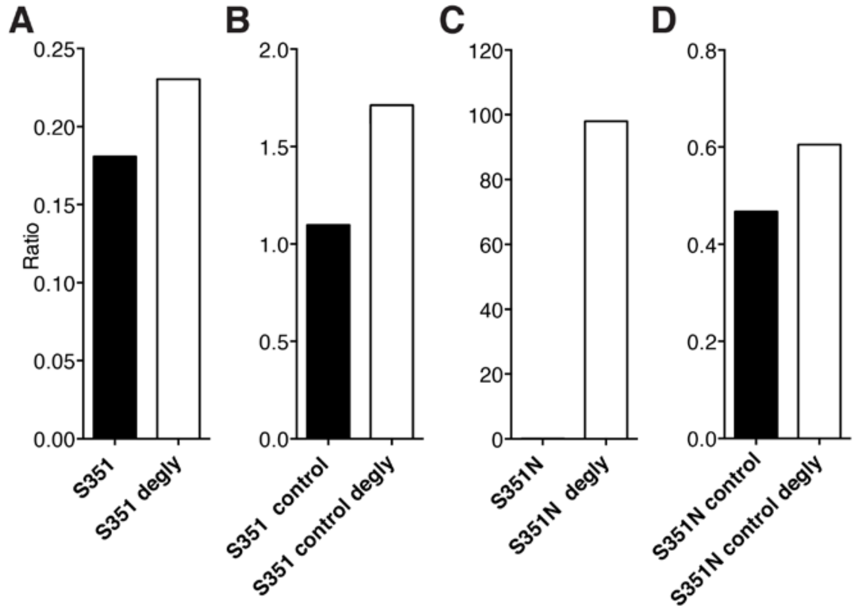

Figure S6, related to Figure 5. The CD6 SNP, S351N introduced an N-linked glycosylation site. Average ratios of the total extracted precursor ion area of peptides from CD6 S351 (A, B) and CD6 S351N (C, D) for S351 (FNNSNLCSQSLAAR) (A), control (B), S351N (FNNSNLCNQSLAAR) (C) and control (D) peptides to precursor ion areas of CD6 control peptides which do not contain N-linked glycosylation sites before (black bars) and after deglycosylation (white bars). The glycosylation site (underlined) at aa 345 (F$\underline{N_{345}NS}$NLCNQSLAAR) in CD6 S351 is not glycosylated.

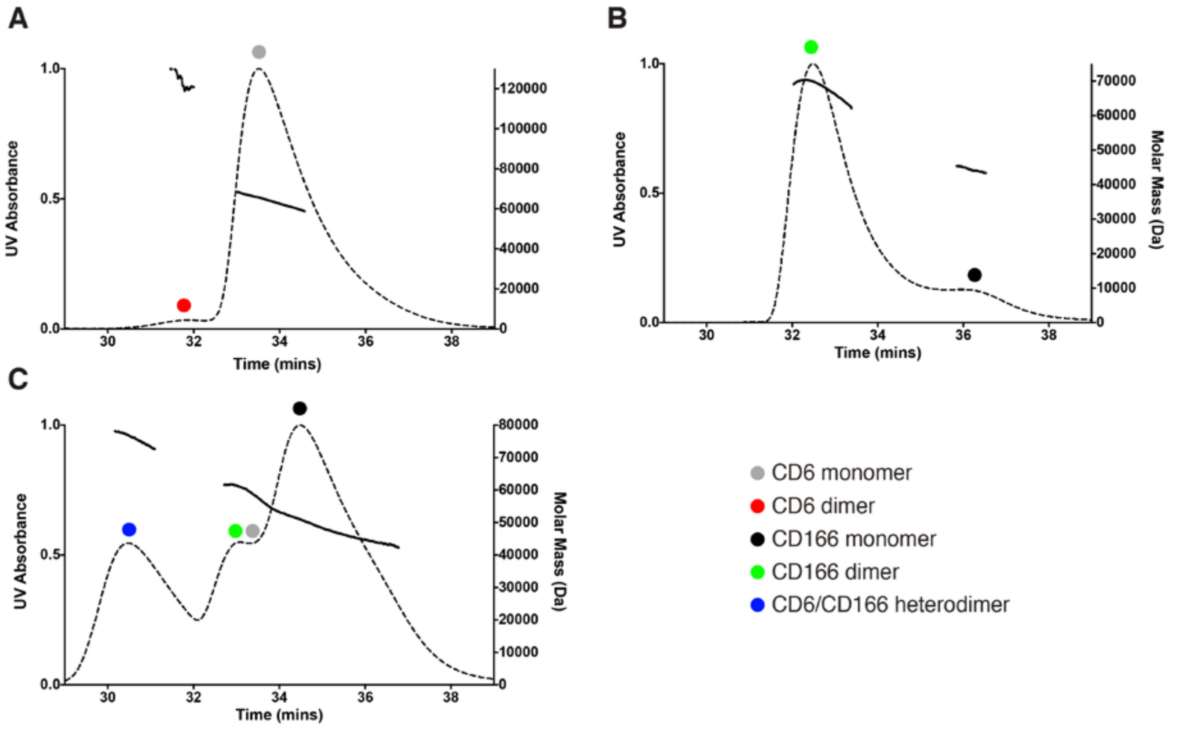

Figure S7, related to Figure 7. MALS showed that CD6 (39 µM) (A) and CD166 VVC (28 µM) (B) are predominantly monomeric and dimeric respectively in solution. Mixing equimolar amounts (27 µM) of CD6 and CD166 VVC (C) altered the species present consistent with formation of heterodimers at the expense of CD6 monomers and CD166 homodimers.

Table S1, related to Figure S2. There is no detectable contribution of CD6 domains 1 and 2 to the interaction with CD166 VVC as measured by SPR at 37ºC. Standard errors of the means were <0.01.

**Molecule immobilised**	**Mean K_D (μM)** ***(range)***	***n***
CD6 d1-3S	0.077 *(0.067 – 0.091)*	3
CD6 d2-3S	0.081 *(0.067 – 0.091)*	3
CD6 d3S	0.086 *(0.077 – 0.097)*	3

Table S2, related to Figures 2 and 5. Residues in CD6 which were mutated to alanine are mapped on the CD6 structure in Figures 2 and 5. (*) = Residues mutated previously to charged residues. For reference, a collated list of the previously reported mutagenesis data is shown (Bodian et al., 1997; Skonier et al., 1997).

Residue	No effect	Disrupted ligand binding	% of WT Binding at K_D ±SEM	*n*
R283*	X		140	1
D291		X	8 ± 4	3
S292		X	37 ± 4	3
E293*		X	15 ± 6	3
Y295		X	4 ± 1	3
E298		X	6 ± 2	3
R314*		X	17 ± 3	3
K316*	X		81 ± 8	3
S323	X		84 ± 13	2
R325	X		88	1
Y327*		X	6 ± 2	3
S329*		X	19 ± 9	3
N331	X		113	1
N346*		X	10 ± 3	3
N348*		X	42 ± 11	3
L349*		X	20 ± 9	3
S351	X		127 ± 6	3
Q352*		X	24 ± 6	3
S353*		X	21 ± 4	3
L354	X		98 ± 23	3

Previously reported mutagenesis data:

Residue	No effect	Disrupted domain structure	Disrupted ligand binding
A271R	X		
Q277R		X	
V285E	X		
W286R		X	
E293R		X	
P296R	X		
Q304R	X		
Q304E	X		
S305R		X	
R314E		X	
S321K	X		
L322R		X	
Y327R		X	
S329R		X	
E333K	X		
N339D	X		
F344R		X	
N345D			X

N346K			X
N348R			X
Q352R			X
S353K		X	
A355D		X	
R357E		X	
S363K		X	

Table S3, related to Figures 2 and 6. Residues in CD166 which were mutated (Skonier et al., 1996a, b) are mapped on the CD166 structure in Figures 2, 5 and 6. (*) = Residues mutated in this study.

Residue	No effect on CD6 binding	Disrupted domain structure	Disrupted CD6 binding	Potential homophilic binding site
Y29A	X			
N32A	X			
I40A		X		
D46A	X			
P48A	X			
N50A	X			
L51A	X			
M52A	X			X
F53A/E			X	X
K55A*/E/D*			X	X
K57A	X			X
E59A	X			
K60A	X			
F67A/E			X	
F70A*/E/Y			X	X
S73A	X			
T74A	X			
K75A/D*			X	
Q79A	X			
Y80A/F	X			
D81A/K*			X	
D82A	X			
E85A	Not expressed			
K87A	X			
R89A	X			
L92A		X		
E94A	X			
S99Y		X		
R104A	X			
R110A	X			
V112A	X			
M114A			X	X
V116Y	X			
T117A			X	
E118A/R*			X	
D119A	X			
N120A	X			
F122A	X			
E123A	X			X
T126Y	X			

Table S4, related to Figure 5 and Figure S6. A CD6 SNP, S351N in the binding site reduced affinity for CD166.

**Molecule immobilised**	**Mean K_D ±SEM (µM)** ***(range)***	**Mean k_{off} ±SEM (s^{-1})** ***(range)***	***n***
S351	0.120 ± 0.016 *(0.089 – 0.144)*	0.072 ± 0.002 *(0.069 – 0.074)*	3
S351N	0.599 ± 0.055 *(0.506 – 0.695)*	0.337 ± 0.030 *(0.280 – 0.384)*	3
S351A	0.083 ± 0.013 *(0.057 – 0.098)*	0.049 ± 0.001 *(0.047 – 0.051)*	3

Table S5, related to Figure 5 and Figure S7. CD6 is glycosylated. [1]Total number of peptide spectral matches (PSM) containing the N glycosylation site before PNGase F treatment. [2]Total number of PSM containing the N glycosylation site after Peptide -N-Glycosidase (PNGase) F treatment. [3]Inconclusive due to low number of peptides containing the N glycosylation site. [4]Although there is an increase of approximately 3 fold more PSM after deglycosylation, there are 43 before, suggesting a partially glycosylated site. [5]Total number of PSM for control peptides containing cysteines before and after deglycosylation.

**Residue**	**N-linked glycosylation site**	**Glycosylation status**	**PSM[1]**	**PSM PNGase F[2]**
28	NTS	YES	1	48
49	NGS	YES	7	91
112	NTS	Inconclusive[3]	0	2
118	NAT	Inconclusive[3]	0	7
229	NCS	YES	1	38
339	NCS	Partial[4]	43	119
345	NNS	NO	162	160
351	NQS	YES	1	80
Cys90[5]	-	-	243	248
Cys170[5]	-	-	51	56

Supplemental Methods Biacore Analysis

Analysis using a BIAcore 3000 was carried out using transiently expressed CD6 and CD166 as fusion proteins with rat CD4 domains 3 and 4 (CD4d3+4) fusion proteins immobilized via a biotinylated tag to streptavidin or rat CD4d3+4 mAb (OX68) (Brown et al., 1998). The CD6 and CD166 fusion proteins were constructed with an XbaI site upstream of the initiation methionine and SalI site (g tcg ac) at the end of the extracellular region in frame with rat CD4d3+4 and all contained the membrane proximal stalk region (Hassan et al., 2004). The same extracellular fragment of CD166 was used to produce soluble CD166d1-5 as a 6 His tagged protein in Lec 3.2.8.1 cells. Similarly to human CD6d3-CD4d3+4, human CD6d2+3-CD4d3+4 was produced from template containing the human CD5 leader (Bowen et al., 1996; Brown et al., 1998). Mutants of CD6 and CD166 were made using a Q5 SDM kit (NEB). K55A, K55D and F70A CD166 mutants were synthesized (Invitrogen). Equilibrium binding and kinetic analyses were carried out in HBS-EP running buffer at 20 µl/min at 37 ºC or 25 ºC as stated in figure legends. Equilibrium binding is plotted after subtraction of the response in the control flow cell and the equilibrium dissociation constant, K_D calculated by curve fitting using a Langmuir 1:1 binding model using Graphpad Prism (one site specific binding). Monoclonal antibodies specific for CD6 domain 1 were MT605 (BD Pharmingen) and MEM98 (abD Serotec), for CD6 domain 3, OX126 (Hassan et al., 2006) and for CD166 (clone 105901, R&D Systems).

Multi-angle Light Scattering (MALS).

Size exclusion chromatography was performed on a Superdex200 10/30 column (GE Healthcare) equilibrated in 50 mM Tris.HCl, pH 7.5, 150 mM NaCl at 0.4 ml/min. The column was followed in-line by a Dawn Heleos-II light scattering detector (Wyatt Technologies) and an Optilab-Rex refractive index monitor (Wyatt Technologies). Molecular mass calculations were performed using ASTRA 5.3.4.14 (Wyatt Technologies) assuming a

dn/dc value of 0.186 ml/g. Samples are diluted approximately 10-fold when applied to the column.

Mass spectrometry analysis of the glycosylation status

CD6-CD4d3+4 S351N transiently expressed in 293T cells was purified with OX68 mAb coupled to Sepharose 4B and elution with 1M Glycine-HCl, pH 2.5. CD6d1-3 His (S351) and CD6-CD4d3+4 S351N, 5-10 µg/sample, were denatured (50mM TCEP-HCl in 8 M urea, 1 h RT), washed with PBS and cysteines alkylated with iodoacetamide (100 µl 20 mM in PBS for 30 minutes at 4 °C). Samples of CD6 S351 and S351N deglycosylated with Peptide -N-Glycosidase (PNGase F) or untreated were digested with trypsin (Metcalfe et al., 2011). After desalting on a C18 micro column, the samples were resuspended in 0.1 % formic acid containing 2 % acetonitrile and analysed on a Ultimate 3000 UHPLC (Dionex) coupled to a QExactive mass spectrometer (Thermo Fisher Scientific). Samples were injected directly on an in-house packed 25 cm C18 (Bishoff 3 µm bead diameter) column in 0.1% formic acid and separated with a gradient of 0.1 % formic acid in acetonitrile, 5-30 % for 90 min, 30 % - 55 % for 20 min and 98 % for 5 min at 300 nl/min. Data were acquired in a data-dependent mode, automatically switching from MS to collision induced dissociation MS/MS on the top 20 most abundant ions with a precursor ion scan range of 350 – 1650 m/z. Full scan MS spectra were acquired at a resolution of 140,000 and MS/MS scans at 17,500 at a target value 3×10^6 and 1×10^5 ions respectively. Dynamic exclusion was enabled with exclusion duration of 40 s.

The data files from all mass spectrometry runs were combined and searched against the human Swiss-Prot database using Peaks 7 proteomics studio. Precursor mass tolerance was 10 ppm and a fragment ion tolerance was 0.01 m/z with up to three missed trypsin cleavage sites per peptide allowed. Variable modifications were defined as deamidation on asparagine and glutamine, oxidation on methionine and carbamidomethylation on cysteine.

De-novo, peaks-db, SPIDER and peaks of post translational modification algorithms were sequentially used to search against a concatenated target/decoy database, providing an empirical false discovery rate (FDR), results are reported at a 1 % target/decoy FDR for both peptides and proteins. 2^+ ions and retention time windows were extracted for the desired peptides (peptide 1 VEMLEHGEWGSVCDDTWDLEDAHVVCR, peptide 2 LVDGGGACAGR, peptide 3 QLGCGWAVQALPGLHFTPGR and peptide 4 GVWNTVCDSEWYPSEAK for non glycosylated control peptides and FNNSNLC(S/N)QSLAAR containing the S351N SNP. Precursor ion areas of the peptides and modified variants such as oxidation of methionine and deamidation were extracted using MS1 filtering in Skyline. The total precursor ion area of each peptide was the sum of all modified variants of that peptide and was used to calculate the following ratios in each sample. 351 peptide:peptide 1, 351 peptide:peptide 2, 351 peptide:peptide 3 and 351 peptide:peptide 4. The following ratios were determined as controls peptide 1:peptide 3, peptide 2:peptide 4, peptide 3:peptide 1 and peptide 4:peptide 2. Averages of the individual 351 and control peptide ratios are reported.

For the other glycosylation sites the total number of peptide spectral matches (PSM) containing each N-glycosylation site before and after deglycosylation with PNGase F were determined from the data produced in the Peaks 7 SPIDER search. As a control the total number of PSM containing Cys90 and Cys70 were determined before and after deglycosylation with PNGase F.

References

Bowen, M.A., Bajorath, J., Siadak, A.W., Modrell, B., Malacko, A.R., Marquardt, H., Nadler, S.G., and Aruffo, A. (1996). The amino-terminal immunoglobulin-like domain of activated leukocyte cell adhesion molecule binds specifically to the membrane-proximal scavenger receptor cysteine-rich domain of CD6 with a 1:1 stoichiometry. J. Biol. Chem. *271*, 17390-17396.

Brown, M.H., Boles, K., van der Merwe, P.A., Kumar, V., Mathew, P.A., and Barclay, A.N. (1998). 2B4, the natural killer and T cell immunoglobulin superfamily surface protein, is a ligand for CD48. J. Exp. Med. *188*, 2083-2090.

Hassan, N.J., Barclay, A.N., and Brown, M.H. (2004). Frontline: Optimal T cell activation requires the engagement of CD6 and CD166. Eur. J. Immunol. *34*, 930-940.

Hassan, N.J., Simmonds, S.J., Clarkson, N.G., Hanrahan, S., Puklavec, M.J., Bomb, M., Barclay, A.N., and Brown, M.H. (2006). CD6 Regulates T-Cell Responses through Activation-Dependent Recruitment of the Positive Regulator SLP-76. Mol. Cell. Biol. *26*, 6727-6738.

Metcalfe, C., Cresswell, P., Ciaccia, L., Thomas, B., and Barclay, A.N. (2011). Labile disulfide bonds are common at the leucocyte cell surface. Open biology *1*, 110010.

Skonier, J.E., Bowen, M.A., Emswiler, J., Aruffo, A., and Bajorath, J. (1996a). Mutational analysis of the CD6 binding site in activated leukocyte cell adhesion molecule. Biochemistry *35*, 14743-14748.

Skonier, J.E., Bowen, M.A., Emswiler, J., Aruffo, A., and Bajorath, J. (1996b). Recognition of diverse proteins by members of the immunoglobulin superfamily: delineation of the receptor binding site in the human CD6 ligand ALCAM. Biochemistry *35*, 12287-12291.
