# Supplementary material for: Structures of CD6 and Its Ligand CD166 Give Insight into Their Interaction
Source: Structure. 2015 Aug 4;23(8):1426–36. doi: 10.1016/j.str.2015.05.019 (PMC4533223; doi:10.1016/j.str.2015.05.019)
Supplement: Document S2. Article plus Supplemental Information [file mmc2.pdf]

Article

Structure

Structures of CD6 and Its Ligand CD166 Give Insight into Their Interaction

Graphical Abstract

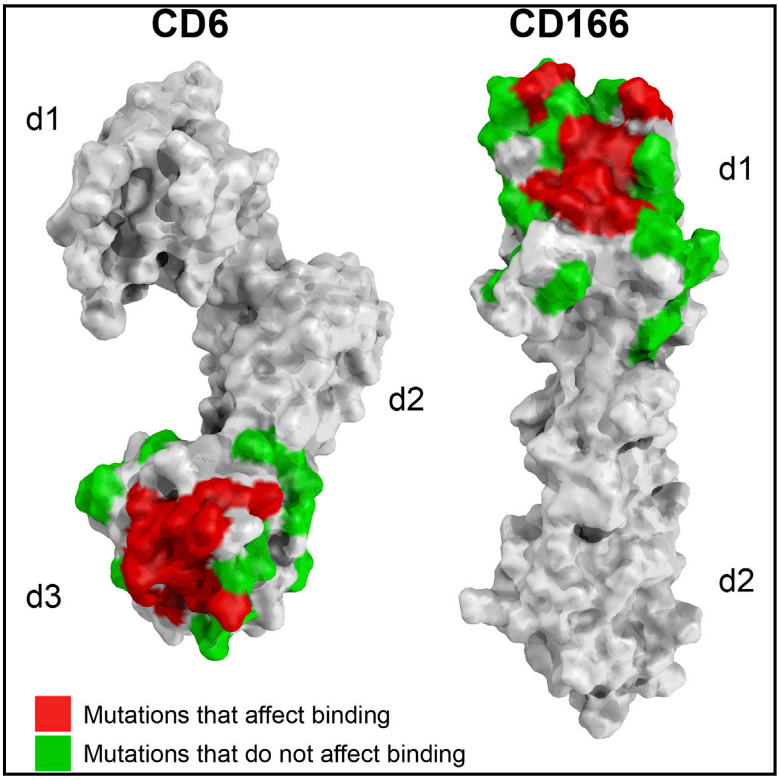

Authors

Paul E. Chappell, Lee I. Garner, Jun Yan, ..., Carol V. Robinson, Susan M. Lea, Marion H. Brown

Correspondence

marion.brown@path.ox.ac.uk

In Brief

Chappell et al. present structures of the T-cell surface receptor, CD6, the first of consecutive scavenger receptor cysteine rich domains and its ligand, CD166. The structures give insight into how CD6 and its interactions are perturbed by competition between homophilic and heterophilic interactions, SNPs, and mAbs.

Highlights

- First structure of consecutive scavenger receptor cysteine rich domains in CD6
- Structure of the two N-terminal domains of CD166 which is the ligand for CD6
- Mapping binding sites on CD6 and CD166
- Insight into how CD6 and its interactions are perturbed by polymorphisms and mAbs

Chappell et al., 2015, Structure *23*, 1426–1436
August 4, 2015 ©2015 The Authors
http://dx.doi.org/10.1016/j.str.2015.05.019

Structure
Article

Structures of CD6 and Its Ligand CD166 Give Insight into Their Interaction

Paul E. Chappell,[1] Lee I. Garner,[1] Jun Yan,[2] Clive Metcalfe,[1] Deborah Hatherley,[1] Steven Johnson,[1] Carol V. Robinson,[2] Susan M. Lea,[1] and Marion H. Brown[1,*]
[1]Sir William Dunn School of Pathology, University of Oxford, South Parks Road, Oxford OX1 3RE, UK
[2]Department of Chemistry, Physical and Theoretical Chemistry Laboratory, University of Oxford, South Parks Road, Oxford OX1 3RE, UK
*Correspondence: marion.brown@path.ox.ac.uk
http://dx.doi.org/10.1016/j.str.2015.05.019
This is an open access article under the CC BY license (http://creativecommons.org/licenses/by/4.0/).

SUMMARY

CD6 is a transmembrane protein with an extracellular region containing three scavenger receptor cysteine rich (SRCR) domains. The membrane proximal domain of CD6 binds the N-terminal immunoglobulin superfamily (IgSF) domain of another cell surface receptor, CD166, which also engages in homophilic interactions. CD6 expression is mainly restricted to T cells, and the interaction between CD6 and CD166 regulates T-cell activation. We have solved the X-ray crystal structures of the three SRCR domains of CD6 and two N-terminal domains of CD166. This first structure of consecutive SRCR domains reveals a nonlinear organization. We characterized the binding sites on CD6 and CD166 and showed that a SNP in CD6 causes glycosylation that hinders the CD6/CD166 interaction. Native mass spectrometry analysis showed that there is competition between the heterophilic and homophilic interactions. These data give insight into how interactions of consecutive SRCR domains are perturbed by SNPs and potential therapeutic reagents.

INTRODUCTION

CD6 is a lymphocyte membrane receptor characterized as being primarily expressed on T cells and some B cells. The extracellular region of CD6 contains three scavenger receptor cysteine rich (SRCR) domains and a membrane proximal stalk (Figure 1). CD6 engages in cell-cell interactions by binding to the immunoglobulin superfamily (IgSF) cell surface receptor, CD166, otherwise known as activated leukocyte adhesion molecule (ALCAM) (Aruffo et al., 1997; Bowen et al., 1995) (Figure 1). CD166 is expressed more widely than CD6 and is found on both hematopoietic and nonhematopoietic cells (Chitteti et al., 2013). In this *trans* interaction between cells, the membrane proximal SRCR domain of CD6 binds the N-terminal domain of CD166 (Aruffo et al., 1997).

In addition to heterophilic interactions with CD6, CD166 also engages in homophilic interactions in *trans* between apposing cells (Te Riet et al., 2007; van Kempen et al., 2001) (Figure 1). As in binding CD6, homophilic interactions of CD166 depend on the N-terminal domain (Bowen et al., 1996; van Kempen et al., 2001). Although weaker than the heterophilic CD6/CD166 interaction (Hassan et al., 2004; Te Riet et al., 2007), CD166 homophilic interactions have pleiotropic effects and regulate not only normal but also neoplastic cells (Chitteti et al., 2013; Weidle et al., 2010). Soluble fusion proteins of the extracellular regions of CD6 or CD166 inhibit T-cell responses (Gimferrer et al., 2004; Hassan et al., 2004; Zimmerman et al., 2006). A CD6 monoclonal antibody (mAb) against domain 3, characterized biochemically as blocking CD6/CD166 interactions distinguished between effects of heterophilic CD6/CD166 and homophilic CD166/CD166 interactions (Hassan et al., 2006). This mAb inhibited antigen-specific proliferation in a polyclonal population of human blood cells, revealing a costimulatory role for engagement of CD6 by CD166 (Hassan et al., 2006). The inhibitory effect of blocking extracellular engagement of CD6 and CD166 identifies these receptors as potential therapeutic targets for immunosuppression.

Costimulation by CD6 is dependent on phosphorylation of a C-terminal tyrosine motif which is specific for the adaptor protein, SLP-76 (Hassan et al., 2006). CD6 has an extraordinarily long cytoplasmic tail (244 amino acids) and provides an alternative to LAT as a scaffold for assemblies of signaling proteins in T cells (Roncagalli et al., 2014). In contrast, CD166 has a short cytoplasmic region that regulates adhesion through a link with the cytoskeleton (Te Riet et al., 2014). Expression of CD6 raises the threshold of activation, and overall net inhibitory or costimulatory effects depend on the particular immune response being measured (Hassan et al., 2006; Oliveira et al., 2012). The dual effects of CD6 make it difficult to interpret the consequences of SNPs that alter the expression of CD6 and correlate with susceptibility to multiple sclerosis (De Jager et al., 2009; Heap et al., 2010; Kofler et al., 2011; Swaminathan et al., 2013).

It is not yet clear how the multiple domains of CD6 are important for function. In the absence of domain 3, no interaction with CD166 can be detected biochemically (Bowen et al., 1996; Hassan et al., 2004). However, CD6 domain 1 mAbs are inhibitory in cellular assays (Kofler et al., 2011; Nair et al., 2010; Singer et al., 1996; Zimmerman et al., 2006), and one has been recently licensed in India for use as an immunosuppressant for psoriasis (Jayaraman, 2013). Inhibition by a CD6 domain 1 mAb depends on the presence of CD6 domain 3, supporting the hypothesis that CD6 domain 1 mAbs perturb CD166/CD6 interactions between cells by steric hindrance (Bowen et al., 1995; Kofler et al., 2011).

Consecutive domains are a feature of group B members of the SRCR domain superfamily (reviewed in Herzig et al., 2010) with three consecutive group B SRCR domains found in the closely

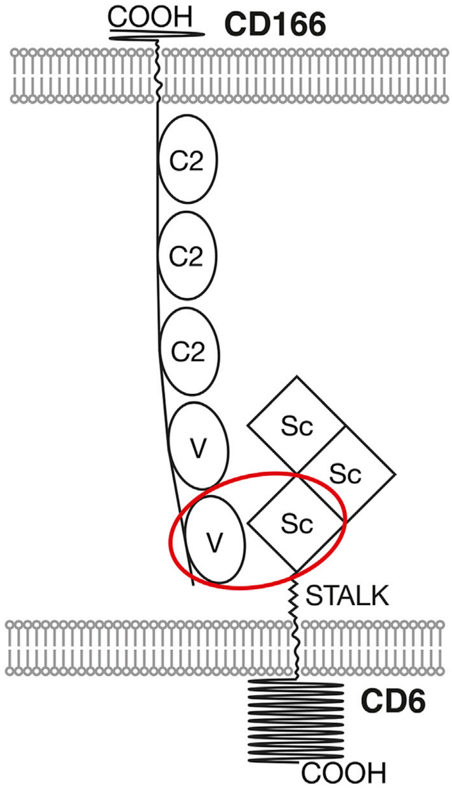

Figure 1. Heterophilic and Homophilic Interactions of CD6 and CD166 between Cells

CD6 contains three SRCR domains (Sc, squares) and CD166 contains five IgSF domains (two V and three C2). The membrane proximal domain of CD6 binds the N-terminal immunoglobulin superfamily (IgSF) domain (oval) of CD166.

related cell surface proteins CD5 and CD6, and the soluble Apoptosis Inhibitor of Macrophages (AIM), also known by other names (Spα, CD5L), which comprises solely three consecutive SRCR domains. The involvement of multiple consecutive SRCR domains in ligand binding has been well characterized for the leukocyte surface receptor CD163, the second and third SRCR domains cooperating in ligand binding to hemoglobin/haptoglobin complexes (Nielsen et al., 2013). All three SRCR domains contribute to ligand binding by AIM (Spα, CD5L), which was found to be associated with immunoglobulin M (IgM) in serum (Arai et al., 2013; Tissot et al., 2002).

The crystal structure of CD6 is the first of consecutive SRCR domains, earlier structures being of single SRCR domains (Garza-Garcia et al., 2008; Hohenester et al., 1999; Rodamilans et al., 2007). We reveal a nonlinear arrangement of the CD6 SRCR domains, which leads us to hypothesize about the roles of the individual domains and their different interactions.

RESULTS

Purification, Characterization, and Crystallization of CD6 and CD166

Recombinant His-tagged proteins of human CD6 SRCR domains 1–3 and human CD166 IgSF domains 1–3, i.e. the two N-terminal V domains and one C domain (CD166 VVC), were stably expressed in Lec 3.2.8.1 Chinese hamster ovary (CHO) cells (Chen and Stanley, 2003), purified, and deglycosylated. Protein crystals of the individual proteins were grown and diffraction data collected. We confirmed that the proteins used in this study interact as expected (data not shown and see Supplemental Information) (Bowen et al., 2000; Hassan et al., 2004).

X-Ray Crystal Structure of CD6 Reveals a Nonlinear Organization of Consecutive SRCR Domains

X-Ray diffraction data were collected to 3.15 Å and the structure solved by molecular replacement using a search model derived from the structure of a single SRCR domain of Mac-2 binding protein (PDB: 1BY2, 26.7% sequence identity to CD6 domain 1, 44.5% to CD6 domain 2, and 26.1% to CD6 domain 3; Hohenester et al., 1999) (Table 1). The X-ray structure of CD6 is the first structure for a protein containing consecutive SRCR domains (Figures 2A and 2B).

The structure reveals a nonlinear organization of consecutive SRCR domains of CD6. Despite the interdomain interactions not being conserved, the orientations of each domain relative to its adjacent domain are very similar, both burying approximately 270 Å^2. A consequence of this orientation at the interfaces between individual SRCR domains is a nonlinear consecutive domain structure, resulting in the three domains spanning 78.1 Å, a shorter distance than if the domains were in a linear arrangement (Figures 2A and 2B). This structural feature was also observed in a lower resolution dataset collected from a different crystal form (data not shown), supporting the idea that this is a biologically relevant structural feature. There is a disordered region in CD6 domain 1 in a similar position to a flexible loop in CD5 domain 1 (Garza-Garcia et al., 2008).

Mapping Domain 3 Mutants on the CD6 Structure Identifies the Ligand Binding Site for CD166

Previous studies identified CD6 domain 3 and CD166 domain 1 as being sufficient to mediate ligand binding (Bowen et al., 1996; Whitney et al., 1995). The role of the other two CD6 SRCR domains in the heterophilic interaction is not clear. We quantified binding of soluble recombinant CD166 VVC to different forms of streptavidin-immobilized chimeric CD6 by surface plasmon resonance (SPR), in an attempt to detect any contribution of CD6 domains 1 and 2. No difference was detected in the binding of CD166 VVC to CD6, CD6 lacking domain 1, or CD6 lacking domains 1 and 2 (Figure S1; Table S1).

Using our structure of CD6 and previous mutagenesis data (Bodian et al., 1997; Skonier et al., 1997), we carried out a more extensive mutagenesis study on CD6 domain 3 (Figures 2B and S2; Table S2; see Figure 4A) to define the residues critical for CD166 binding. These data were mapped onto the structure, clearly identifying and defining the boundaries of the surface of CD6 that interacts with CD166. There is a high degree of amino acid conservation in this binding face between human, mouse, and rat CD6 sequences (Figure 3). Within this region amino acids have been identified that reduce binding to CD166, so it is likely that different species use the same face (Bowen et al., 2000) (Figure 3). We extended our previously published cross-species binding analysis to include rat

Table 1. Crystallographic Data for CD6 and CD166

	CD6	CD166
Data Collection		
X-Ray source	Diamond Light Source, beamline i04-1	Diamond Light Source, beamline i04-1
Wavelength (Å)	0.92	0.92
Resolution range (Å)	77.93–3.15 (3.23–3.15)	51.14–1.86 (1.91–1.86)
Space group	P 63 2 2	P 43 21 2
Unit Cell		
a, b, c (Å)	161.48, 161.48, 93.85	72.32, 72.32, 105.04
α, β, γ (°)	90, 90, 120	90, 90, 90
Total reflections	69,680 (5,176)	313,66 (22,938)
Unique reflections	12,884 (933)	24,110 (1,731)
Multiplicity	5.4 (5.5)	13.0 (13.3)
Completeness (%)	99.6 (99.8)	99.9 (99.9)
Mean $I/\sigma(I)$	7.2 (2.2)	20.1 (3.8)
R_{merge} (all I^+ and I^-) (%)	23.2 (80.0)	8.0 (65.9)
Refinement		
Resolution range (Å)	77.93–3.15	21.09–1.86
No. of reflections (work/test set)	12,883/629	24,029/1,229
No. of atoms (protein, glycans, ligands, waters)	4,399, 27, 60, 43	3,496, 54, 160, 186
Protein residues	324	218
Mean B-factors (protein, glycans, ligands, waters) (Å^2)	37.85, 61.24, 35.33, 10.28	32.53, 47.28, 59.05, 38.80
R_{work} (%)	25.31	22.17
R_{free} (%)	28.31	23.91
Rmsd from ideal values (bonds, Å)	0.0061	0.0061
Rmsd from ideal values (angles, °)	0.76	0.93
Ramachandran plot		
Favored regions (%)	95.05	96.79
Outliers (%)	0	0

Rmsd, root-mean-square deviation.

CD6, and showed that human CD166 bound human, mouse, and rat CD6 with the same affinity (Hassan et al., 2006 and data not shown).

Mapping Domain 1 Mutants on the X-Ray Crystal Structure of CD166 Identifies the Ligand Binding Site for CD6

X-Ray diffraction data for crystals containing CD166 VVC were collected to 1.86 Å and the structure solved by molecular replacement with a search model derived from the second IgSF domain of RAGE (PDB: 3CJJ, 16.4% sequence identity to domain 1 and 30.3% to domain 2; Koch et al., 2010). Only the first two domains could be resolved in the electron density map (Figures 2C and 2D). The crystal lattice did not contain space to accommodate the third domain, suggesting it had been proteolytically cleaved prior to crystal growth.

We mapped previous mutagenesis data that defined residues in CD166 domain 1 critical for CD6 binding (Skonier et al., 1996a, 1996b) onto the crystal structure (Figures 2D; Table S3; see Figure 4B). This confirmed the prediction that binding to CD6 is mediated by the AGFCC′C″ face, in common with many other IgSF interactions (Skonier et al., 1996a, 1996b). We repeated mutagenesis of five of these residues in CD166 domain 1 and confirmed that they all disrupted CD6/CD166 interactions (Figure S3; Table S3).

There Is Complementary Electrostatic Potential between CD166 and CD6 Binding Sites

Mutagenesis of CD6 domain 3 (Bodian et al., 1997; Skonier et al., 1997) (Table S2 and Figure 4A: N346K, N348R, Q352R) showed that altering the charge from negative to positive in CD6 inhibited CD166 binding. Conversely, altering the charge from positive to negative in CD166 domain 1 (Table S3 and Figure 4B: K55E) inhibited binding to CD6 (Skonier et al., 1996a, 1996b). Loss of charged amino acids (Table S3 and Figure 4B: E118A, K75A, D81A) in CD166 domain 1 also reduced binding, consistent with electrostatic potential being important for ligand binding. The calculated electrostatic potentials of the proposed interface between CD6 and CD166 are compatible with their interaction. The CD6 surface features a stripe of negative charge flanked with positive charge, and the CD166 surface features a complementary positively charged stripe flanked by negative charge (Figure 4).

Mapping Domain 1 mAb Epitopes on the CD6 Structure Shows How Ligand Binding between Cells Might Be Disrupted

Inhibition by CD6 domain 1 mAbs has only been observed between cells, suggesting that intermembrane dimensions are important for their effects (Bowen et al., 1996, 1995). To gain some insight from the CD6 structure as to how CD6 domain 1 mAbs might disrupt CD6/CD166 interactions, we mapped the epitope of a CD6 domain 1 mAb, MT605, which has been shown to inhibit immune responses between cells expressing CD6 and CD166 (Kofler et al., 2011; Singer et al., 1996; Zimmerman et al., 2006). To identify the epitope, we produced alanine mutants of a number of surface-exposed residues in CD6 domain 1. Binding of MT605 was abolished by R77A (Figure S4). Binding of another CD6 domain 1 mAb, MEM-98, was unaffected by R77A but was eliminated by E63A. MT605 was unaffected by E63A (Figure S4). In the orientation shown in Figure 2, R77 lies at the top of CD6 domain 1. A large antibody binding in this position may prevent the close approach between cell membranes needed for CD6/CD166 engagement.

CD6 Nonsynonymous SNPs Associated with Multiple Sclerosis which Alter Expression Are Buried

Of the SNPs identified in CD6, five alter amino acids in domains 2 and 3 (Figure 5). Homozygotes for two of these SNPs (R225W and A257V) in domain 2 have been associated with susceptibility to multiple sclerosis (Swaminathan et al., 2013). These two residues are buried within the domain and may disrupt the domain structure and stability of the molecule, explaining the correlation

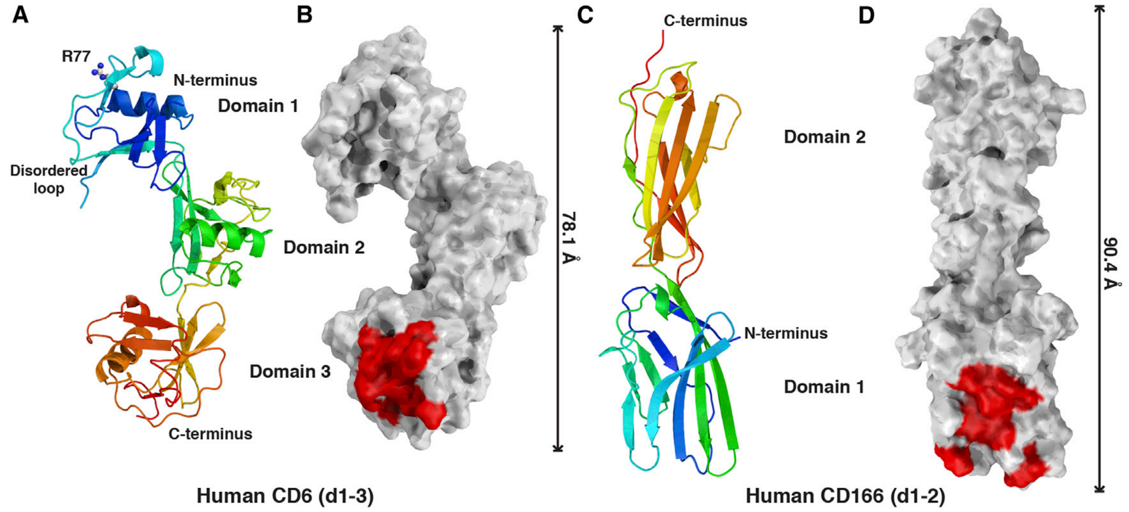

Figure 2. Structures of CD6 and CD166 Identify a Binding Interface and Reveal a Nonlinear Arrangement of Domains in CD6
(A and C) Cartoon diagrams colored from blue at the N terminus to red at the C terminus.
(B and D) Surface representations, with mutations in CD6 affecting ligand binding colored red.
Similar interdomain orientations of CD6 domains result in an overall nonlinear organization. A disordered loop exists in the N-terminal domain of CD6. Binding of CD6 domain 1 mAb MT605 was specifically reduced by R77A mutation (see Figure S4).

between reduced expression and susceptibility to multiple sclerosis.

The CD6 SNP, S351N, Results in Glycosylation that Disrupts CD6/CD166 Interactions

Of the surface-exposed SNPs in CD6, the change from S351 to S351N found in 10% of the genomes analyzed (http://www.1000genomes.org) is the only residue close to the ligand binding site. We mutated CD6 S351 to S351N and compared the binding of soluble CD166 VVC to the two variants by SPR. CD166 VVC bound with a 10-fold weaker affinity to CD6 S351N compared with CD6 S351 (Figure S5; Table S5). Analysis of the kinetics of binding showed an increase in the dissociation rate of CD6 S351N compared with CD6 S351 (Figure S5; Table S4).

As S351N introduces a consensus N-linked glycosylation site (NQS), binding of CD166 may be sterically hindered by carbohydrate. There are seven consensus N-glycosylation sites in the three extracellular domains of CD6. In the recombinant protein, we observed electron density for GlcNAc attached to residue N229 in the second domain. There was no observable electron density for the other potential glycosylation sites in CD6. Mass spectrometry was used to determine whether recombinant CD6 S351N is glycosylated. As glycosylated peptides are not identified by liquid chromatography-tandem mass spectrometry

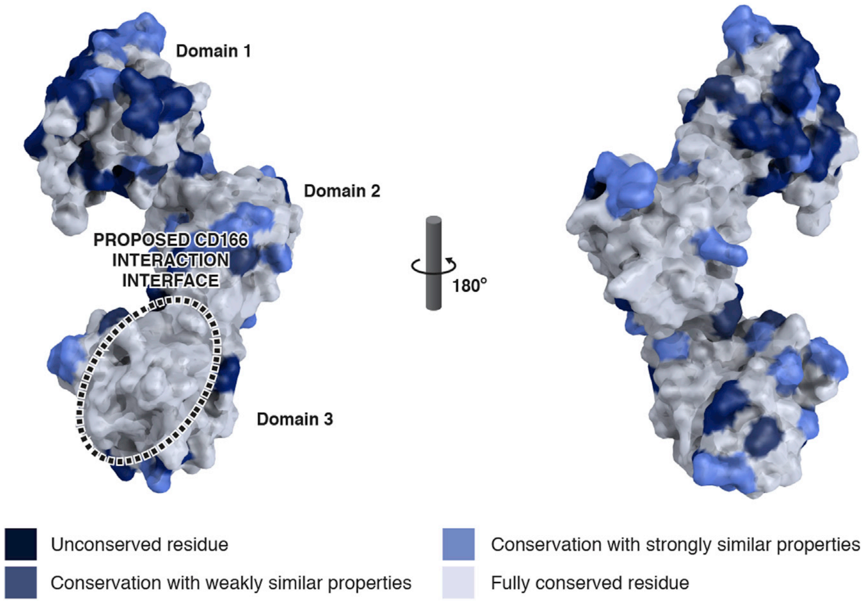

Figure 3. CD6 SRCR Domains, Residues 43–364, which Bind CD166 Are Conserved Across Species
Human (UniProt: P30203), mouse (UniProt: Q61003), and rat (UniProt: Q5FVU4) CD6 bind human CD166 (Hassan et al., 2006 and data not shown). Compared with CD6 SRCR domain 3, which binds CD166, CD6 domains 1 and 2 have more variation in amino acid sequence between human, mouse, and rat CD6.

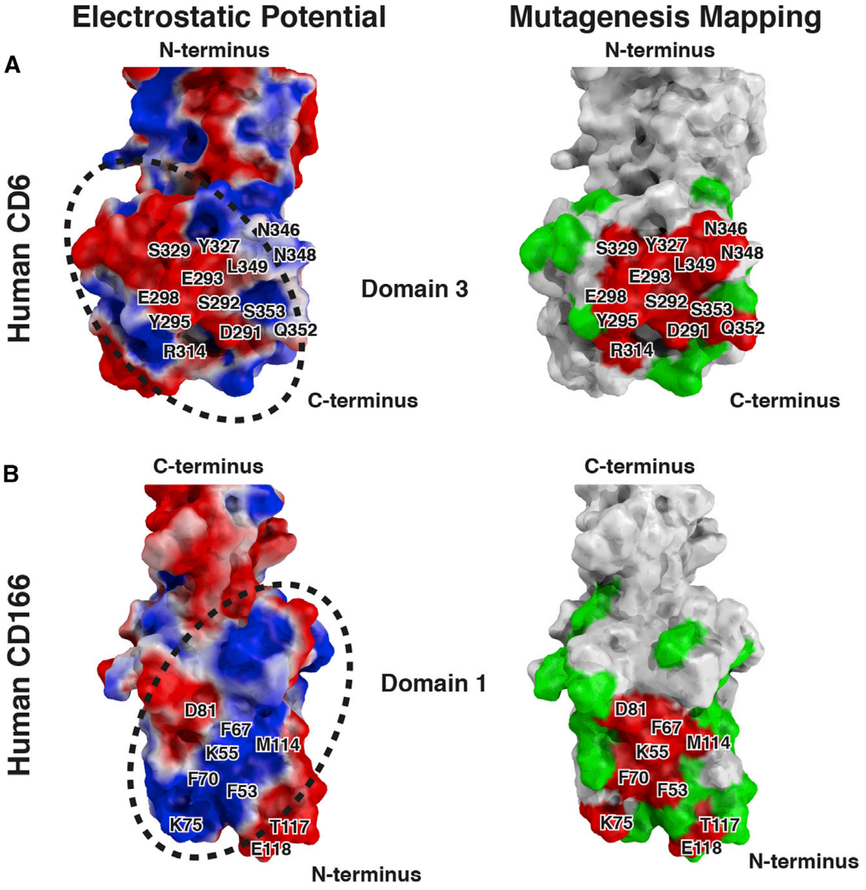

Figure 4. Complementary Electrostatic Potential between CD166 and CD6 Binding Faces

Surface representations of human CD6 (A) and human CD166 (B) displaying electrostatic potential (negative charge in red and positive charge in blue) and mutations that do not affect binding (green) and that affect binding (red). Areas of complementary electrostatic potential are outlined with a dotted line.

due to the unknown mass of the N-linked carbohydrate, we quantified the precursor ion intensity obtained from extracted ion chromatograms of the FNNSNLCS$_{351}$QSLAAR and FNNSNLCN$_{351}$QSLAAR tryptic peptides before and after deglycosylation (Figure S6; Table S5). The S351 peptide was not glycosylated (Figures S6A and S6B), whereas the ion intensity of the S351N peptide was dependent on deglycosylation, showing that it was glycosylated (Figures S6C and S6D). Consistent with the interpretation that S351N disrupted ligand binding by introducing a bulky carbohydrate moiety in proximity to the CD166 binding site, the CD6 mutant S351A restored binding, as measured by SPR (Figure S5; Table S5).

The glycosylation states of the other putative glycosylation sites in CD6 were determined qualitatively from the mass spectrometry data by comparing the number of peptide spectral matches of peptides containing each N-linked glycosylation site before and after deglycosylation with peptide-*N*-glycosidase F, showing that N28, N49, N229, and N351 are glycosylated and that N339 appears to be partially glycosylated (Table S5).

Heterodimers Are Formed at the Expense of CD6 and CD166 Homodimers

Homophilic and heterophilic interactions of CD166 are mediated by the N-terminal domain. Mutations in the A′GFCC′C″ face, which disrupt CD6/CD166 interactions (Figure 2), map to the crystal contacts between the two N-terminal domains in the CD166 structure (Figure 6) (Bowen et al., 2000).

If the heterophilic and homophilic binding sites overlap, as implied by the CD166 crystal contacts, homophilic CD166 interactions would be disrupted by the formation of heterodimers with CD6. We examined the molecular species present when CD6 and CD166 were mixed in comparison with the individual components by native mass spectrometry. When CD6 and CD166 were analyzed separately, we found that both CD6 and CD166 were predominantly in monomer-dimer equilibrium (Figures 7A and 7B). When CD6 and CD166 were mixed, there was an increase in the relative abundance of heterodimers, a corresponding decrease of the CD6 and CD166 monomers, and a complete depletion of homodimers when compared with the unmixed samples (Figure 7C). These data show that heterodimers are formed at the expense of CD6 and CD166 homodimers. Due to the heterogeneity in molecular masses caused by glycosylation, the collision voltage on the mass spectrometer was increased to achieve more accurate mass determination, and these experimentally calculated masses are shown in Table 2. The mass of the heterodimer was confirmed by analysis of its composition by tandem mass spectrometry (data not shown).

Comparison of the molecular species identified in native mass spectrometry and multi-angled light scattering also revealed a dynamic equilibrium between the different species (Figure S7).

DISCUSSION

The structures of CD6 and CD166 give insight into the heterophilic CD6/CD166 and homophilic CD166/CD166 interactions and those of other proteins containing consecutive SRCR domains. The structure of CD6 is the first for proteins containing consecutive SRCR domains. One striking feature is the nonlinear domain organization, which has implications for the topology and orientations of interactions with individual domains.

The Role of Consecutive SRCR Domains in CD6/CD166 Interactions

The relatively high affinity *trans* interaction between CD6 and CD166 on apposing cells is mediated by the CD6 membrane proximal domain. The main contribution of the other two CD6

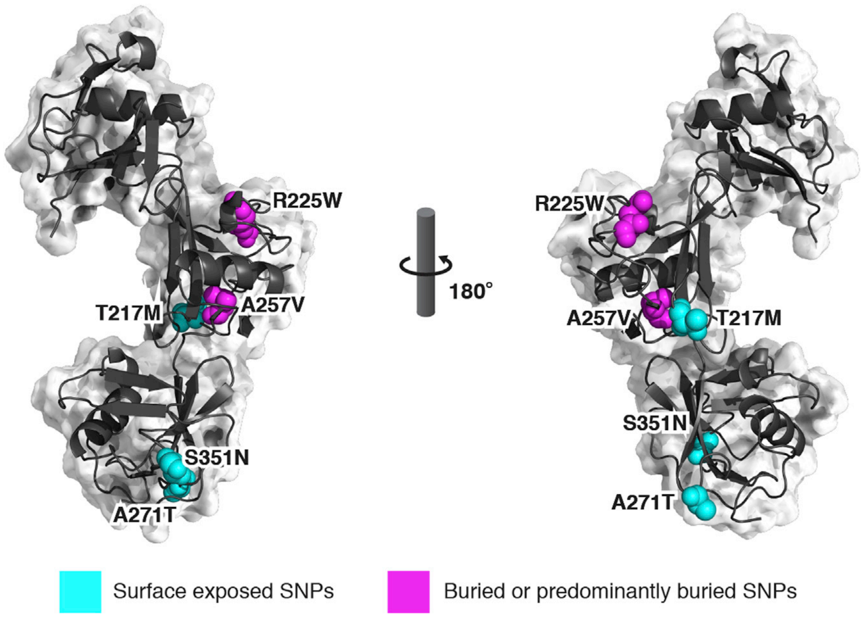

Figure 5. Nonsynonymous SNPs in CD6 Associated with Disease Are Not in the CD166 Binding Site

SNPs are mapped on the structure of CD6 and displayed as spheres.

SRCR domains to the CD6/CD166 interaction may be to regulate accessibility of CD166 to the membrane proximal domain of CD6, as formation of CD6/CD166 heterodimers involves competition with the homophilic CD166 interactions. *Trans* CD166 homophilic interactions were inhibited by preventing clustering in *cis* on the cell surface with an antibody specific for the membrane proximal domains of CD166 or by a dominant negative mutant lacking the N-terminal domain (van Kempen et al., 2001, 2004; Weidle et al., 2010). Weak interactions in *cis* between the membrane proximal domains of CD166 at the cell surface may enhance homophilic adhesion in *trans* (van Kempen et al., 2001; Wu et al., 2011). The projection of CD6 domain 1, which results from the nonlinear domain organization, may be important for preventing clustering of CD166 at the cell surface, and may provide an additional steric hindrance mechanism to disrupt the CD166 *trans* homodimers between cells.

The topology of the CD6 SRCR domains shows how CD6 domain 1 mAbs might impede access of CD166 to the membrane proximal domain of CD6 between cells (Bowen et al., 1995; Kofler et al., 2011). Inhibition of the CD6/CD166 interaction by CD6 domain 1 mAbs has only been observed between cells and is likely to be dependent on the level of CD6/CD166 engagement (Bowen et al., 1995; Castro et al., 2007; Kofler et al., 2011; Singer et al., 1996; Zimmerman et al., 2006).

The main role of domain 2 of CD6 may be as a spacer to maintain the projection of CD6 domain 1. However, in the characterization of an interaction between the SRCR domains of the closely related protein CD5 and the V region of the heavy chain of immunoglobulin, first reported in rabbit, only the isolated domain 2 of human CD5 was capable of binding the V region of the heavy chain of human immunoglobulin (Pospisil et al., 2000). CD5 differs in topology from CD6 in containing a proline-rich linker between domains 1 and 2 that may be important for accessibility to domain 2.

A subsidiary role for domains other than CD6 domain 3 and CD166 domain 1 in CD6/CD166 interactions, which are below the detection limit of SPR analysis, cannot be completely ruled out, as there are data suggesting weak interactions of other domains in high-avidity binding assays (Bowen et al., 1996). In the context of a lattice formation between cells (Weidle et al., 2010), a very low-affinity interaction may be productive (Wu et al., 2011). CD6 domain 2 may be important for divalent cation-dependent interactions of CD6, as it contains a conserved triacidic motif D187/D188/E253 (Nielsen et al., 2013). CD6/CD166 interactions occur in the absence of divalent cations (Hassan et al., 2004; Patel et al., 1995; L.I.G., unpublished data).

Homodimers of CD166 and also CD6 were detected in native mass spectrometry (Figure 7). When mixed, the main species was heterodimers of CD6 and CD166. Based on data for CD5, CD6 homodimers are more likely to form in *cis* on the same cell surface (Bamberger et al., 2011; Brown and Lacey, 2010). The nonlinear arrangement of the SRCR domains in CD6 is compatible with individual domains engaging in interactions in different orientations. If the angle of projection of CD6 from the cell surface is as depicted in Figures 1 and 2, it places the N-terminal domain in a position that could be important for creating contacts on the same cell surface, including homophilic interactions. Deletion of the N-terminal domain of CD6 resulted in a protein that was not stable at the cell surface, whereas CD6 containing domain 1 but lacking the membrane proximal domain is expressed stably at the cell surface (Castro et al., 2007; Kofler et al., 2011; M.H.B., unpublished data). Dimerization of CD6 might be important for stabilization at the surface in the absence of ligand binding, and ligand engagement by CD166 may be important for stabilizing CD6 monomers at the cell surface to maintain immune responses (Zimmerman et al., 2006).

Heterophilic CD6/CD166 and Homophilic CD166/CD166 Interactions

In the competition between CD6 domain 3 and the N-terminal domain of CD166, heterodimers are formed at the expense of the weaker homophilic interactions of CD166 (Hassan et al., 2004; Te Riet et al., 2007; and see above). Crystal contacts in the CD166 structure suggest that the sites for heterophilic and homophilic binding on CD166 domain 1 overlap (Figure 6). Alternatively, heterophilic and homophilic interactions may compete through steric hindrance.

The structures of the interacting domains, the membrane proximal domain of CD6, domain 3, and the N-terminal domain of CD166 showed that mutants that disrupt binding to CD166 cluster together on the surface of CD6 (Bodian et al., 1997; Skonier et al., 1997), identifying the binding face on domain 3.

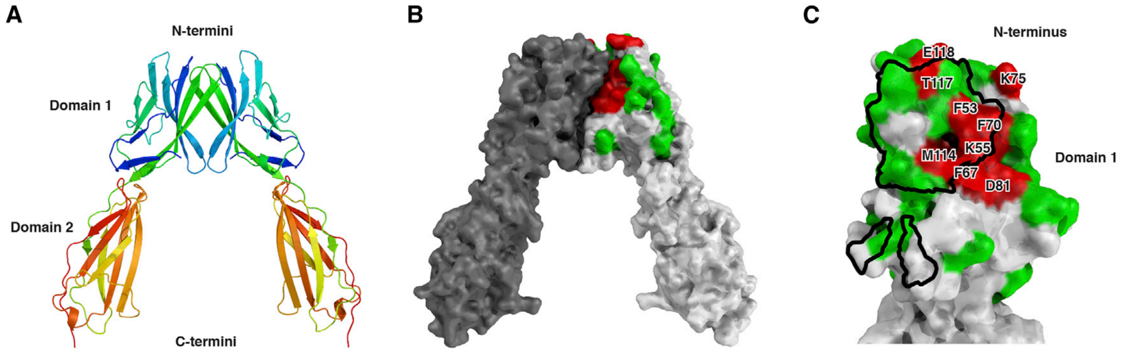

Figure 6. Crystal Contacts Identify a Potential CD166 Domain 1 Homophilic Binding Face

Cartoon representations colored from blue at the N-terminus to red at the C-terminus (A) and surface representations (B) of CD166 domains 1 and 2, showing a binding interface between the A′GFCC′C″ faces (Bowen et al., 2000). The homophilic interface is outlined in black (C).

Complementary mapping of mutants of CD166 that affected ligand binding delineated the binding site predicted to be on the A′GFCC′C″ face of the CD166 structure (Bodian et al., 1997; Skonier et al., 1997).

The binding faces of CD6 SRCR domain 3 and CD166 domain 1 are predominantly positively and negatively charged, respectively (Figure 4) (Bowen et al., 2000). Ligand binding by SRCR domains in MARCO and CD163, in groups A and B, respectively, involves complementarity in charge (Nielsen et al., 2013; Ojala et al., 2007). Electrostatic potential is likely to be important in the CD6/CD166 interaction.

Implications for Other Proteins Containing Consecutive SRCR Domains

The interaction between CD6 and CD166 functions in the adaptive immune system, whereas the majority of SRCR superfamily domains are found in the innate immune system (Herzig et al., 2010). Preservation of the three consecutive SRCR domains may be important for maintaining more promiscuous low-affinity interactions (Abdi et al., 2014; Arai et al., 2013) with ligand binding depending on avidity with contributions from each domain, as has been observed in the interaction between AIM (Spα, CD5L) and IgM (Arai et al., 2013). There may be a similar nonlinear domain arrangement in other proteins containing consecutive SRCR domains that is important for multiple domains making contact with a ligand. Modeling the conformation of longer arrays of SRCR domains with similar length linker sequences suggests that these proteins would form a helical structure.

Group A SRCR domains containing proteins that consist of polypeptides with single SRCR domains trimerize, which may create an alternative topology for multivalent binding. There is a model based on a crystal structure for how membrane distal single SRCR domains of MARCO multimerize to form a large ligand binding face for pathogens (Ojala et al., 2007). Direct pathogen binding has been described for several SRCR domains, including CD6 (Sarrias et al., 2007). A bacterial binding peptide motif was first identified in an SRCR domain of DMBT1, otherwise known as salivary agglutinin (Bikker et al., 2004; Madsen et al., 2010). A consensus sequence for this bacterial binding peptide is found in CD6 SRCR domain 2 (Bikker et al., 2004). Examining the position of this peptide on the structure of CD6, the region is predominantly surface exposed and is on the opposite face to the CD166 binding site. Its significance is uncertain. Mutation of this consensus sequence in the SRCR domain protein WC1 did not affect bacterial binding (Hsu et al., 2015).

Effects of Single Polymorphisms in CD6

Selection pressure for nonsynonymous SNPs in CD6 domain 2 that are associated with susceptibility to multiple sclerosis (Figure 5) may simply be to reduce expression levels (Swaminathan et al., 2013). Reduced expression levels will lower both the threshold of activation and ligand engagement (Hassan et al., 2006). The strongest association of a SNP in CD6 with disease susceptibility is in an intron, and correlates with a reduced ratio of expression of full-length CD6 compared with a form of CD6 which lacks the ligand binding domain, suggesting that the dominant effect is mediated by decreased ligand binding (Castro et al., 2007; Kofler et al., 2011). Disease association for the less common SNP, S351N, which has reduced affinity for CD166, has not yet been reported. Mutagenesis and mass spectrometric analysis provided evidence that reduced binding was due to glycosylation of CD6 at S351N. We assume this high frequency SNP is likely to be associated with subtle functional changes in CD6 activity.

Topology of CD6/CD166 Interactions

On engagement by CD166, CD6 has been observed to move into areas of close contact between T cells and antigen-presenting cells (Castro et al., 2007; Gimferrer et al., 2004; Zimmerman et al., 2006), suggesting that the interacting receptors will match the dimensions of the T-cell receptor and other receptors observed to colocalize, ~140 Å (Dushek et al., 2012) (Figure 2). The nonlinear domain structure may be important for accommodating CD6 in areas of close apposition between cells. The nonlinear structure of the three SRCR domains spans 78.1 Å. This excludes the 37 amino acid membrane proximal stalk region of unknown dimensions. The two IgSF V domains of CD166 span 90.4 Å, comparable with the dimensions of the two IgSF V domains in another cell surface receptor, JAM (86 Å, PDB: 1F97). Extrapolating from the dimensions of the two CD166 IgSF V and three C domains taking the size of a C domain to

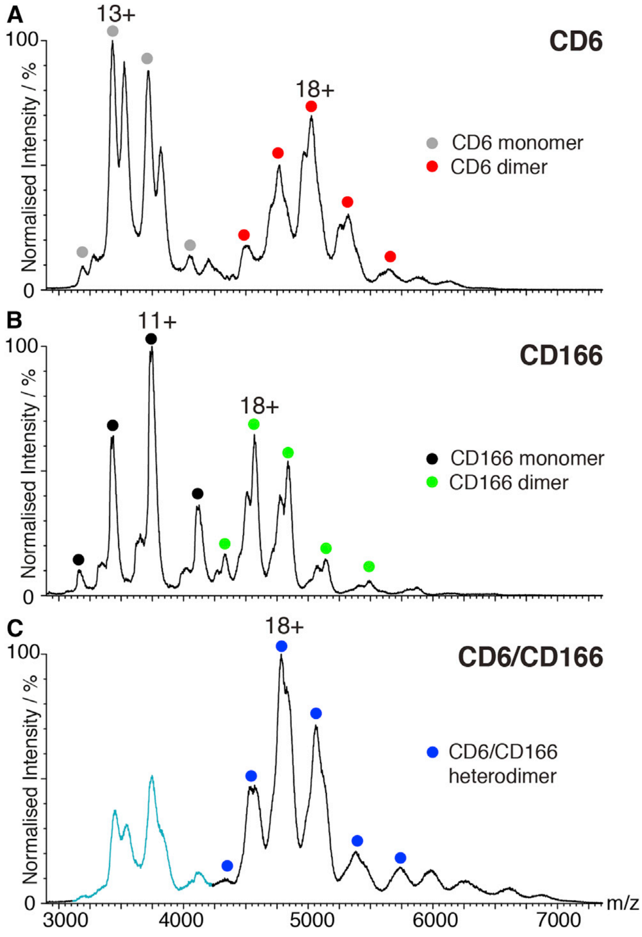

Figure 7. Heterodimers Are Formed at the Expense of CD6 and CD166 Homodimers

Native mass spectrometry data are shown for the CD6 (A) and CD166 (B) proteins separately, and for the CD6/CD166 mixture (C). The CD6 and CD166 proteins exist predominantly in a monomer-dimer equilibrium. Charge state series are indicated with circles: gray and red for CD6 monomer and dimer; and black and green for CD166 monomer and dimer, respectively. In the mixture (C), the CD6/CD166 heterodimer, indicated with blue circles, is the most abundant species. Smaller amounts of individual CD6 and CD166 monomers, colored in cyan, also exist. Where more than one mass was found for a species due to glycosylation, only the most abundant one is indicated on the spectra for clarity. All experimentally calculated masses can be found in Table 2.

be 35 Å from human CD2 domain 2 (PDB: 1HNG), the five domains in a linear array would be predicted to span ~200 Å. This suggests there is flexibility and/or deviation from the perpendicular by CD166 between apposing cells to optimize formation of *trans* interactions, as has been proposed for five domain E-cadherins (Wu et al., 2011). If the parallel, as distinct from anti-parallel orientation of the interacting CD166 N-terminal domains, is physiological, it will restrict the dimensions of homophilic *trans* interactions (Te Riet et al., 2014; van Kempen et al., 2001). Determination of the X-ray crystal structures of CD6 and CD166 allows us to create a more accurate model of how these proteins regulate interactions at the cell surface, and to design the most effective therapeutic reagents.

Table 2. Calculated Mass Species of CD6 and CD166 from Native Mass Spectrometry

	Experimentally Calculated Mass (Da)	Standard Deviation (Da)
CD6		
Monomer 1	44,509	±3
Monomer 2	45,721	±4
Dimer 1	89,135	±13
Dimer 2	90,173	±16
Dimer 3	91,436	±13
CD166		
Monomer 1	39,751	±8
Monomer 2	40,139	±12
Monomer 3	40,975	±4
Monomer 4	41,198	±6
Dimer 1	80,045	±9
Dimer 2	81,046	±24
Dimer 3	82,112	±24
CD6/CD166		
Dimer 1	84,557	±40
Dimer 2	85,592	±32
Dimer 3	86,770	±24

EXPERIMENTAL PROCEDURES

Recombinant Proteins for Crystallization

Three human CD6 (GenBank: U34623; UniProt: P30203) SRCR domains (residues 1–364) and two V-like domains and one C-like domain (VVC) (residues 1–335) of human CD166 (UniProt: Q13740) were amplified from plasmid DNA (Bowen et al., 1996) and expressed as endoglycosidase H sensitive His-tagged proteins (CD6; LCSASRGHHHHHH CD166; YLDLSTRHHHHHH) containing their native leader sequences using PEE14 vector in CHO Lec 3.2.8.1 cells (Chen and Stanley, 2003). CHO cell lines selected for stable expression were grown in cell factories to confluence; sodium butyrate (2 mM) was then added and the cells were allowed to secrete for 2–3 weeks before harvesting. Tissue culture supernatant was concentrated (Sartorius Vivablock, molecular weight cutoff 10 kDa) and proteins purified using nickel chromatography columns (NiNTA, Qiagen) and elution with an imidazole gradient in 10 mM HEPES, 150 mM NaCl (pH 7.4). Proteins were immediately dialyzed to remove imidazole and then subjected to size-exclusion chromatography (Superdex200 column; GE Healthcare). For crystallization, proteins were deglycosylated with EndoHf (New England Biolabs), 0.3–2.5 U/ml in 10 mM HEPES, 150 mM NaCl (pH 7.4) at 37°C for 2 hr and analyzed by SDS-PAGE.

Crystallization

The three extracellular SRCR domains of human CD6 were crystallized using the sitting drop method in 0.1 M ammonium sulfate, 0.3 M sodium formate, 0.1 M sodium cacodylate, 3% w/v PGA-LM, 20% MPD (pH 6.5) at 20°C. Drops were set up using 2.5 μl of a protein solution containing a complex of CD6 and CD166 at an OD_{280} of 2.22, and 2.5 μl of the reservoir solution. CD6 crystals grew within 4 weeks.

The two membrane distal immunoglobulin domains of human CD166 were crystallized using the sitting drop method in 0.1 M sodium HEPES (pH 7.5) and 25% w/v PEG-2000 MME. Drops were set up using 2 μl of a protein solution containing CD166 domains 1–3 at an OD_{280} of 4.58. Crystals grew after 4 weeks. Crystals were flash-frozen in liquid nitrogen and data were collected at the Diamond Light Source, Harwell, at 100 K on beamline i04-1.

Data Collection and Processing

Native datasets were collected for CD6 and CD166, to 3.15 Å and 1.86 Å, respectively. The data were integrated and scaled using XDS (Kabsch, 2010)

and aimless (Evans and Murshudov, 2013) as implemented in XIA2 (Winter et al., 2013). Molecular replacement search models were generated using CHAINSAW (Stein, 2008), and large loops removed. The structure of human CD6 was solved by searching for three copies of a search model derived from the Mac2 binding protein, a single SRCR domain (PDB: 1BY2; Hohenester et al., 1999) using PHASER (McCoy et al., 2007), part of the CCP4 software suite (Winn et al., 2011). The human CD166 structure was solved by searching with a search model derived from the second immunoglobulin domain in the ligand binding domain of human RAGE (PDB: 3CJJ; Koch et al., 2010). Model building and refinement were carried out using Coot (Emsley et al., 2010) and AUTOBUSTER (Bricogne et al., 2011). The CD6 and CD166 structures were refined to R_{work}/R_{free} of 0.25/0.28 and 0.22/0.24, respectively. Coordinates were deposited in the PDB (PDB: 5a2e for CD6 and PDB: 5a2f for CD166).

Native Mass Spectrometry

Purified CD6 and CD166, along with a mixture of both proteins, were subjected to native mass spectrometry analysis. Proteins were at a concentration of 20 μM and the mixture formed by incubating an equal amount of CD6 and CD166 (both at 20 μM) for 30 min at room temperature. Samples were buffer exchanged from 10 mM HEPES, 150 mM NaCl (pH 7.4) into 250 mM ammonium acetate (pH 7.6) using Biospin-6 columns (Bio-Rad). Mass spectrometry measurements were carried out on a modified Synapt G1 HDMS (Waters Corp.) Q-ToF mass spectrometer (Bush et al., 2010). The instrument was mass calibrated using a solution of 10 mg/ml cesium iodide in water. Aliquots (2 μl) of samples were delivered to the mass spectrometer by means of nano-electrospray ionization via gold-coated capillaries, prepared in-house (Hernandez and Robinson, 2007). Instrumental parameters were as follows: source pressure 5.6 mbar, capillary voltage 1.20 kV, cone voltage 50 V, trap energy 10 V, bias voltage 5 V, and trap pressure 1.63×10^{-2} mbar. The trap energy was increased to 30 V to obtain more accurate mass measurements.

SUPPLEMENTAL INFORMATION

Supplemental Information includes Supplemental Methods, seven figures, and five tables and can be found with this article online at http://dx.doi.org/10.1016/j.str.2015.05.019.

AUTHOR CONTRIBUTIONS

P.E.C. purified and crystallized protein, solved the human CD6 and CD166 X-ray crystal structures, made the figures, and contributed to writing of the manuscript. L.I.G. purified protein and performed SPR experiments. J.Y., C.V.R., and C.M. carried out native and glycosylation mass spectrometry experiments, respectively. D.H. produced cell lines. S.M.L. and S.J. solved the human CD6 and CD166 X-ray crystal structures. M.H.B. directed the project and wrote the manuscript.

ACKNOWLEDGMENTS

This work was supported by a Medical Research Council, UK Programme Grant (G0400808). P.E.C. was funded by a BBSRC D.Phil. studentship (BB/F016085/1). S.J. is funded by an MRC Programme Grant (MR/M011984/1) to S.M.L. and work in S.M.L.'s laboratory is supported by a Wellcome Trust Investigator Award (100298/Z/12/Z). We thank Neil Barclay for critical reading of the manuscript.

Received: September 9, 2014
Revised: May 18, 2015
Accepted: May 21, 2015
Published: July 2, 2015

REFERENCES

Abdi, K., Singh, N.J., Spooner, E., Kessler, B.M., Radaev, S., Lantz, L., Xiao, T.S., Matzinger, P., Sun, P.D., and Ploegh, H.L. (2014). Free IL-12p40 monomer is a polyfunctional adaptor for generating novel IL-12-like heterodimers extracellularly. J. Immunol. *192*, 6028–6036.

Arai, S., Maehara, N., Iwamura, Y., Honda, S., Nakashima, K., Kai, T., Ogishi, M., Morita, K., Kurokawa, J., Mori, M., et al. (2013). Obesity-associated autoantibody production requires AIM to retain the immunoglobulin M immune complex on follicular dendritic cells. Cell Rep. *3*, 1187–1198.

Aruffo, A., Bowen, M.A., Patel, D.D., Haynes, B.F., Starling, G.C., Gebe, J.A., and Bajorath, J. (1997). CD6-ligand interactions: a paradigm for SRCR domain function? Immunol. Today *18*, 498–504.

Bamberger, M., Santos, A.M., Goncalves, C.M., Oliveira, M.I., James, J.R., Moreira, A., Lozano, F., Davis, S.J., and Carmo, A.M. (2011). A new pathway of CD5 glycoprotein-mediated T cell inhibition dependent on inhibitory phosphorylation of Fyn kinase. J. Biol. Chem. *286*, 30324–30336.

Bikker, F.J., Ligtenberg, A.J., End, C., Renner, M., Blaich, S., Lyer, S., Wittig, R., van't Hof, W., Veerman, E.C., Nazmi, K., et al. (2004). Bacteria binding by DMBT1/SAG/gp-340 is confined to the VEVLXXXXW motif in its scavenger receptor cysteine-rich domains. J. Biol. Chem. *279*, 47699–47703.

Bodian, D.L., Skonier, J.E., Bowen, M.A., Neubauer, M., Siadak, A.W., Aruffo, A., and Bajorath, J. (1997). Identification of residues in CD6 which are critical for ligand binding. Biochemistry *36*, 2637–2641.

Bowen, M.A., Patel, D.D., Li, X., Modrell, B., Malacko, A.R., Wang, W.C., Marquardt, H., Neubauer, M., Pesando, J.M., Francke, U., et al. (1995). Cloning, mapping, and characterization of activated leukocyte-cell adhesion molecule (ALCAM), a CD6 ligand. J. Exp. Med. *181*, 2213–2220.

Bowen, M.A., Bajorath, J., Siadak, A.W., Modrell, B., Malacko, A.R., Marquardt, H., Nadler, S.G., and Aruffo, A. (1996). The amino-terminal immunoglobulin-like domain of activated leukocyte cell adhesion molecule binds specifically to the membrane-proximal scavenger receptor cysteine-rich domain of CD6 with a 1:1 stoichiometry. J. Biol. Chem. *271*, 17390–17396.

Bowen, M.A., Aruffo, A.A., and Bajorath, J. (2000). Cell surface receptors and their ligands: in vitro analysis of CD6-CD166 interactions. Proteins *40*, 420–428.

Bricogne, G., Blanc, E., Brandl, M., Flensburg, C., Keller, P., Paciorek, W., Roversi, P., Sharff, A., Smart, O.S., Vonrhein, C., and Womack, T.O. (2011). BUSTER Version 2.10.1.

Brown, M.H., and Lacey, E. (2010). A ligand for CD5 is CD5. J. Immunol. *185*, 6068–6074.

Bush, M.F., Hall, Z., Giles, K., Hoyes, J., Robinson, C.V., and Ruotolo, B.T. (2010). Collision cross sections of proteins and their complexes: a calibration framework and database for gas-phase structural biology. Anal. Chem. *82*, 9557–9565.

Castro, M.A., Oliveira, M.I., Nunes, R.J., Fabre, S., Barbosa, R., Peixoto, A., Brown, M.H., Parnes, J.R., Bismuth, G., Moreira, A., et al. (2007). Extracellular isoforms of CD6 generated by alternative splicing regulate targeting of CD6 to the immunological synapse. J. Immunol. *178*, 4351–4361.

Chen, W., and Stanley, P. (2003). Five Lec1 CHO cell mutants have distinct Mgat1 gene mutations that encode truncated N-acetylglucosaminyltransferase I. Glycobiology *13*, 43–50.

Chitteti, B.R., Bethel, M., Kacena, M.A., and Srour, E.F. (2013). CD166 and regulation of hematopoiesis. Curr. Opin. Hematol. *20*, 273–280.

De Jager, P.L., Jia, X., Wang, J., de Bakker, P.I., Ottoboni, L., Aggarwal, N.T., Piccio, L., Raychaudhuri, S., Tran, D., Aubin, C., et al. (2009). Meta-analysis of genome scans and replication identify CD6, IRF8 and TNFRSF1A as new multiple sclerosis susceptibility loci. Nat. Genet. *41*, 776–782.

Dushek, O., Goyette, J., and van der Merwe, P.A. (2012). Non-catalytic tyrosine-phosphorylated receptors. Immunol. Rev. *250*, 258–276.

Emsley, P., Lohkamp, B., Scott, W.G., and Cowtan, K. (2010). Features and development of Coot. Acta Crystallogr. D Biol. Crystallogr. *66*, 486–501.

Evans, P.R., and Murshudov, G.N. (2013). How good are my data and what is the resolution? Acta Crystallogr. D Biol. Crystallogr. *69*, 1204–1214.

Garza-Garcia, A., Esposito, D., Rieping, W., Harris, R., Briggs, C., Brown, M.H., and Driscoll, P.C. (2008). Three-dimensional solution structure and

conformational plasticity of the N-terminal scavenger receptor cysteine-rich domain of human CD5. J. Mol. Biol. *378*, 129–144.

Gimferrer, I., Calvo, M., Mittelbrunn, M., Farnos, M., Sarrias, M.R., Enrich, C., Vives, J., Sanchez-Madrid, F., and Lozano, F. (2004). Relevance of CD6-mediated interactions in T cell activation and proliferation. J. Immunol. *173*, 2262–2270.

Hassan, N.J., Barclay, A.N., and Brown, M.H. (2004). Frontline: optimal T cell activation requires the engagement of CD6 and CD166. Eur. J. Immunol. *34*, 930–940.

Hassan, N.J., Simmonds, S.J., Clarkson, N.G., Hanrahan, S., Puklavec, M.J., Bomb, M., Barclay, A.N., and Brown, M.H. (2006). CD6 regulates T-cell responses through activation-dependent recruitment of the positive regulator SLP-76. Mol. Cell. Biol. *26*, 6727–6738.

Heap, G.A., Yang, J.H., Downes, K., Healy, B.C., Hunt, K.A., Bockett, N., Franke, L., Dubois, P.C., Mein, C.A., Dobson, R.J., et al. (2010). Genome-wide analysis of allelic expression imbalance in human primary cells by high-throughput transcriptome resequencing. Hum. Mol. Genet. *19*, 122–134.

Hernandez, H., and Robinson, C.V. (2007). Determining the stoichiometry and interactions of macromolecular assemblies from mass spectrometry. Nat. Protoc. *2*, 715–726.

Herzig, C.T., Waters, R.W., Baldwin, C.L., and Telfer, J.C. (2010). Evolution of the CD163 family and its relationship to the bovine gamma delta T cell co-receptor WC1. BMC Evol. Biol. *10*, 181.

Hohenester, E., Sasaki, T., and Timpl, R. (1999). Crystal structure of a scavenger receptor cysteine-rich domain sheds light on an ancient superfamily. Nat. Struct. Biol. *6*, 228–232.

Hsu, H., Chen, C., Nenninger, A., Holz, L., Baldwin, C.L., and Telfer, J.C. (2015). WC1 is a hybrid gammadelta TCR coreceptor and pattern recognition receptor for pathogenic bacteria. J. Immunol. *194*, 2280–2288.

Jayaraman, K. (2013). Biocon's first-in-class anti-CD6 mAb reaches the market. Nat. Biotechnol. *31*, 1062–1063.

Kabsch, W. (2010). XDS. Acta Crystallogr. D Biol. Crystallogr. *66*, 125–132.

Koch, M., Chitayat, S., Dattilo, B.M., Schiefner, A., Diez, J., Chazin, W.J., and Fritz, G. (2010). Structural basis for ligand recognition and activation of RAGE. Structure *18*, 1342–1352.

Kofler, D.M., Severson, C.A., Mousissian, N., De Jager, P.L., and Hafler, D.A. (2011). The CD6 multiple sclerosis susceptibility allele is associated with alterations in CD4+ T cell proliferation. J. Immunol. *187*, 3286–3291.

Madsen, J., Mollenhauer, J., and Holmskov, U. (2010). Review: Gp-340/DMBT1 in mucosal innate immunity. Innate Immun. *16*, 160–167.

McCoy, A.J., Grosse-Kunstleve, R.W., Adams, P.D., Winn, M.D., Storoni, L.C., and Read, R.J. (2007). Phaser crystallographic software. J. Appl. Crystallogr. *40*, 658–674.

Nair, P., Melarkode, R., Rajkumar, D., and Montero, E. (2010). CD6 synergistic co-stimulation promoting proinflammatory response is modulated without interfering with the activated leucocyte cell adhesion molecule interaction. Clin. Exp. Immunol. *162*, 116–130.

Nielsen, M.J., Andersen, C.B., and Moestrup, S.K. (2013). CD163 binding to haptoglobin-hemoglobin complexes involves a dual-point electrostatic receptor-ligand pairing. J. Biol. Chem. *288*, 18834–18841.

Ojala, J.R., Pikkarainen, T., Tuuttila, A., Sandalova, T., and Tryggvason, K. (2007). Crystal structure of the cysteine-rich domain of scavenger receptor MARCO reveals the presence of a basic and an acidic cluster that both contribute to ligand recognition. J. Biol. Chem. *282*, 16654–16666.

Oliveira, M.I., Goncalves, C.M., Pinto, M., Fabre, S., Santos, A.M., Lee, S.F., Castro, M.A., Nunes, R.J., Barbosa, R.R., Parnes, J.R., et al. (2012). CD6 attenuates early and late signaling events, setting thresholds for T-cell activation. Eur. J. Immunol. *42*, 195–205.

Patel, D.D., Wee, S.F., Whichard, L.P., Bowen, M.A., Pesando, J.M., Aruffo, A., and Haynes, B.F. (1995). Identification and characterization of a 100-kD ligand for CD6 on human thymic epithelial cells. J. Exp. Med. *181*, 1563–1568.

Pospisil, R., Silverman, G.J., Marti, G.E., Aruffo, A., Bowen, M.A., and Mage, R.G. (2000). CD5 is A potential selecting ligand for B-cell surface immunoglobulin: a possible role in maintenance and selective expansion of normal and malignant B cells. Leuk. Lymphoma *36*, 353–365.

Rodamilans, B., Munoz, I.G., Bragado-Nilsson, E., Sarrias, M.R., Padilla, O., Blanco, F.J., Lozano, F., and Montoya, G. (2007). Crystal structure of the third extracellular domain of CD5 reveals the fold of a group B scavenger cysteine-rich receptor domain. J. Biol. Chem. *282*, 12669–12677.

Roncagalli, R., Hauri, S., Fiore, F., Liang, Y., Chen, Z., Sansoni, A., Kanduri, K., Joly, R., Malzac, A., Lahdesmaki, H., et al. (2014). Quantitative proteomics analysis of signalosome dynamics in primary T cells identifies the surface receptor CD6 as a Lat adaptor-independent TCR signaling hub. Nat. Immunol. *15*, 384–392.

Sarrias, M.R., Farnos, M., Mota, R., Sanchez-Barbero, F., Ibanez, A., Gimferrer, I., Vera, J., Fenutria, R., Casals, C., Yelamos, J., et al. (2007). CD6 binds to pathogen-associated molecular patterns and protects from LPS-induced septic shock. Proc. Natl. Acad. Sci. USA *104*, 11724–11729.

Singer, N.G., Richardson, B.C., Powers, D., Hooper, F., Lialios, F., Endres, J., Bott, C.M., and Fox, D.A. (1996). Role of the CD6 glycoprotein in antigen-specific and autoreactive responses of cloned human T lymphocytes. Immunology *88*, 537–543.

Skonier, J.E., Bowen, M.A., Emswiler, J., Aruffo, A., and Bajorath, J. (1996a). Mutational analysis of the CD6 binding site in activated leukocyte cell adhesion molecule. Biochemistry *35*, 14743–14748.

Skonier, J.E., Bowen, M.A., Emswiler, J., Aruffo, A., and Bajorath, J. (1996b). Recognition of diverse proteins by members of the immunoglobulin superfamily: delineation of the receptor binding site in the human CD6 ligand ALCAM. Biochemistry *35*, 12287–12291.

Skonier, J.E., Bodian, D.L., Emswiler, J., Bowen, M.A., Aruffo, A., and Bajorath, J. (1997). Mutational analysis of the CD6 ligand binding domain. Prot. Eng. *10*, 943–947.

Stein, N. (2008). CHAINSAW: a program for mutating pdb files used as templates in molecular replacement. J. Appl. Crystallogr. *41*, 641–643.

Swaminathan, B., Cuapio, A., Alloza, I., Matesanz, F., Alcina, A., Garcia-Barcina, M., Fedetz, M., Fernandez, O., Lucas, M., Orpez, T., et al. (2013). Fine mapping and functional analysis of the multiple sclerosis risk gene CD6. PLoS One *8*, e62376.

Te Riet, J., Zimmerman, A.W., Cambi, A., Joosten, B., Speller, S., Torensma, R., van Leeuwen, F.N., Figdor, C.G., and de Lange, F. (2007). Distinct kinetic and mechanical properties govern ALCAM-mediated interactions as shown by single-molecule force spectroscopy. J. Cell Sci. *120*, 3965–3976.

Te Riet, J., Helenius, J., Strohmeyer, N., Cambi, A., Figdor, C.G., and Muller, D.J. (2014). Dynamic coupling of ALCAM to the actin cortex strengthens cell adhesion to CD6. J. Cell Sci. *127*, 1595–1606.

Tissot, J.D., Sanchez, J.C., Vuadens, F., Scherl, A., Schifferli, J.A., Hochstrasser, D.F., Schneider, P., and Duchosal, M.A. (2002). IgM are associated to Sp alpha (CD5 antigen-like). Electrophoresis *23*, 1203–1206.

van Kempen, L.C., Nelissen, J.M., Degen, W.G., Torensma, R., Weidle, U.H., Bloemers, H.P., Figdor, C.G., and Swart, G.W. (2001). Molecular basis for the homophilic activated leukocyte cell adhesion molecule (ALCAM)-ALCAM interaction. J. Biol. Chem. *276*, 25783–25790.

van Kempen, L.C., Meier, F., Egeblad, M., Kersten-Niessen, M.J., Garbe, C., Weidle, U.H., Van Muijen, G.N., Herlyn, M., Bloemers, H.P., and Swart, G.W. (2004). Truncation of activated leukocyte cell adhesion molecule: a gateway to melanoma metastasis. J. Invest. Dermatol. *122*, 1293–1301.

Weidle, U.H., Eggle, D., Klostermann, S., and Swart, G.W. (2010). ALCAM/CD166: cancer-related issues. Cancer Genomics Proteomics *7*, 231–243.

Whitney, G.S., Starling, G.C., Bowen, M.A., Modrell, B., Siadak, A.W., and Aruffo, A. (1995). The membrane-proximal scavenger receptor cysteine-rich domain of CD6 contains the activated leukocyte cell adhesion molecule binding site. J. Biol. Chem. *270*, 18187–18190.

Winn, M.D., Ballard, C.C., Cowtan, K.D., Dodson, E.J., Emsley, P., Evans, P.R., Keegan, R.M., Krissinel, E.B., Leslie, A.G., McCoy, A., et al. (2011). Overview of the CCP4 suite and current developments. Acta Crystallogr. D Biol. Crystallogr. *67*, 235–242.

Winter, G., Lobley, C.M., and Prince, S.M. (2013). Decision making in xia2. Acta Crystallogr. D Biol. Crystallogr. *69*, 1260–1273.

Wu, Y., Vendome, J., Shapiro, L., Ben-Shaul, A., and Honig, B. (2011). Transforming binding affinities from three dimensions to two with application to cadherin clustering. Nature *475*, 510–513.

Zimmerman, A.W., Joosten, B., Torensma, R., Parnes, J.R., van Leeuwen, F.N., and Figdor, C.G. (2006). Long-term engagement of CD6 and ALCAM is essential for T-cell proliferation induced by dendritic cells. Blood *107*, 3212–3220.

Structure, Volume *23*

Supplemental Information

Structures of CD6 and Its Ligand CD166 Give Insight into Their Interaction

Paul E. Chappell, Lee I. Garner, Jun Yan, Clive Metcalfe, Deborah Hatherley, Steven Johnson, Carol V. Robinson, Susan M. Lea, and Marion H. Brown

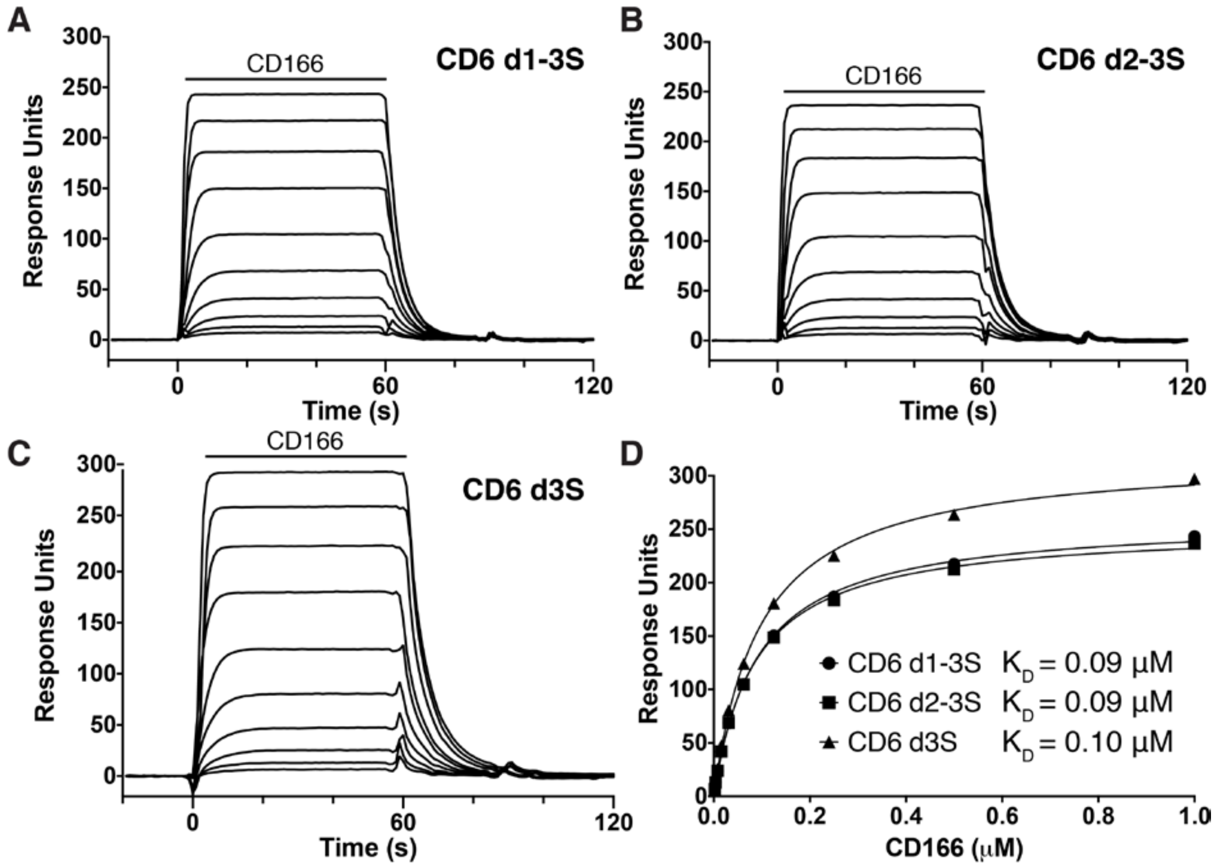

Figure S1 related to Figure 2. There is no detectable contribution of CD6 domains 1 and 2 to the interaction with CD166 as measured by SPR at 37 ºC. Increasing concentrations of soluble CD166 VVC were injected at 37 ºC, over CD6 domains 1-3 (A), CD6 domains 2-3 (B) or CD6 domain 3 (C), plus stalk (S) and rat CD4 d3+4 in a control flow cell immobilised via the biotin tag to streptavidin. (A-C) Sensorgram traces with response over control flow cell subtracted are shown. Bars represent injection period. (D) Specific binding at equilibrium was calculated by subtracting the response over rat CD4 d3+4. The equilibrium dissociation constants, K_D, were calculated by curve fitting (Table S1). Top protein concentration: CD166, 1.0 µM; plus nine two-fold serial dilutions were used for the equilibrium binding experiments.

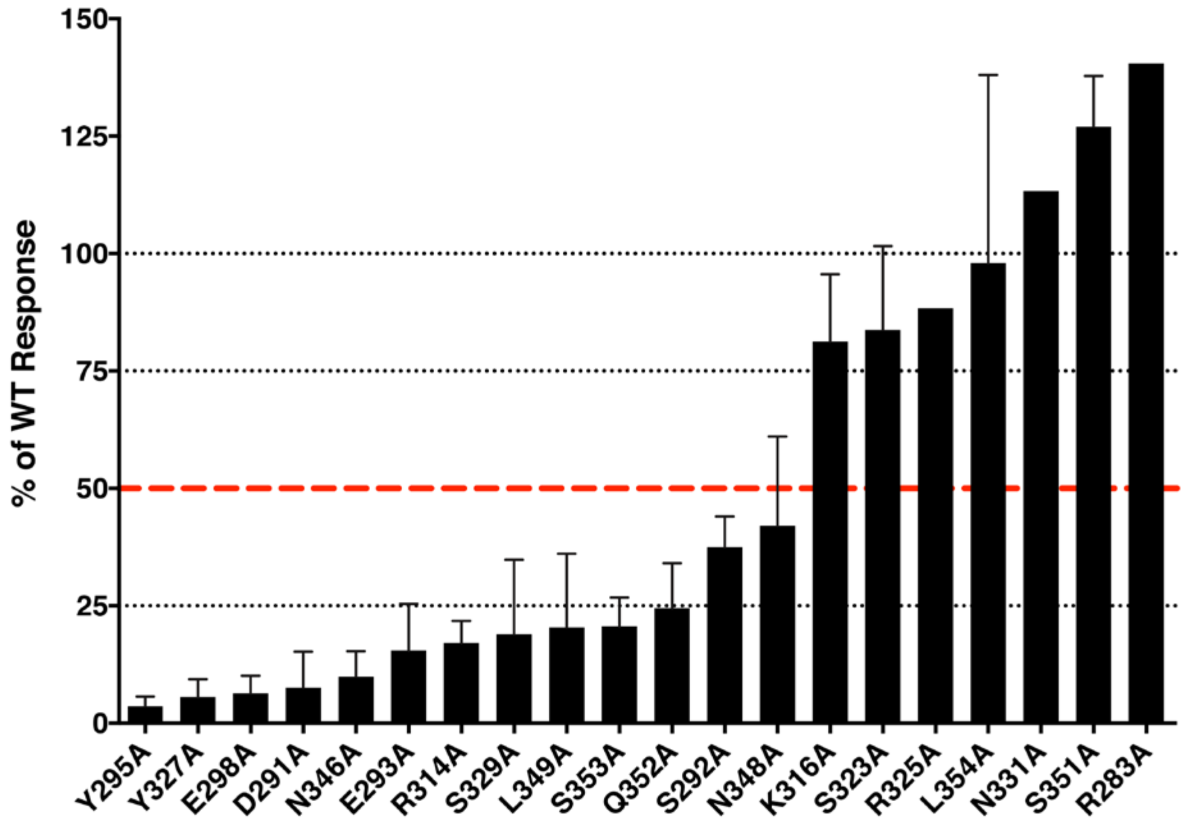

Figure S2, related to Figure 2. Mutation of residues in CD6 domain 3 disrupts CD6/CD166 interactions. Twenty surface exposed residues in CD6 domain 3 were mutated to alanine. Soluble CD166d1-5 at approximately the K_D (0.1 µM) was injected at 25^{0}C, over CD6-CD4d3+4 chimeras immobilised via OX68 mAb. Equilibrium binding data for the mutants are plotted as the mean percentage (±S.E.M) of wild type response normalised to 100 %. The dashed line indicates 50 % response which was used to define residue involvement in binding. There were equivalent levels of all proteins immobilised as monitored by binding of a CD6 domain 3 mAb (OX126).

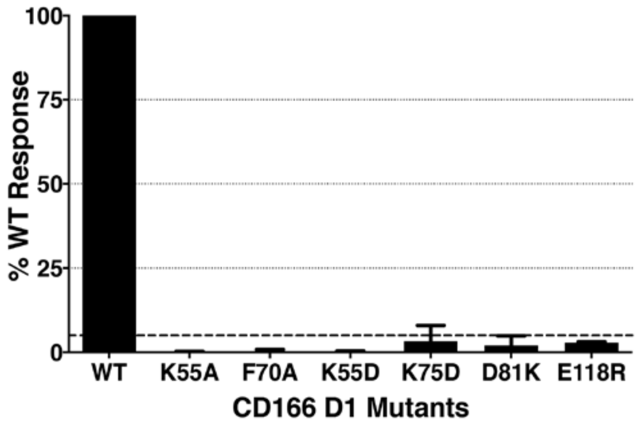

Figure S3, related to Figure 2. Mutation of residues in the CD166/CD166 crystal contacts disrupt CD6/CD166 interactions. Five surface exposed residues in CD166 domain 1 were mutated. Soluble CD6 His at approximately the K_D (0.1µM) was injected at 25^0C, over CD166-CD4d3+4 chimeras immobilised via OX68 antibody. Equilibrium binding data for the mutants are plotted as the mean percentage (±S.E.M) of wild type (WT) response normalised to 100 %. Each mutant reduced binding by ≥ 95% (dashed line). There were equivalent levels of all proteins immobilised as monitored by binding of a CD166 mAb. Expression levels of K55A and K55D were low as previously reported (Skonier et al., 1996a).

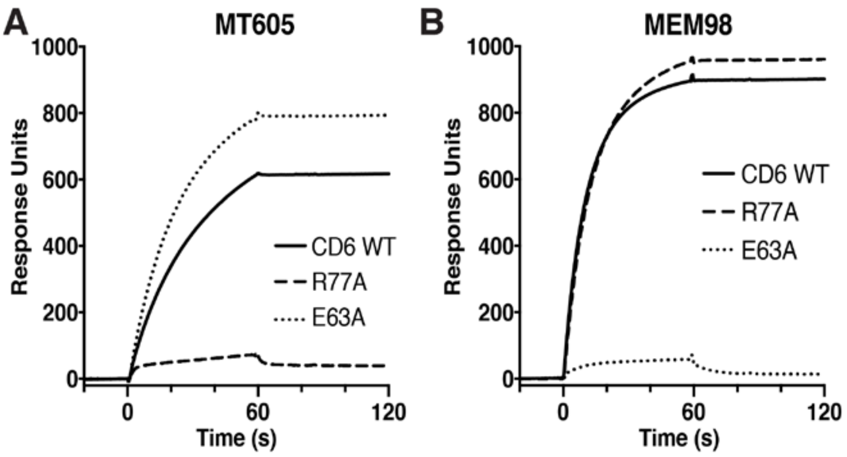

Figure S4, related to Figure 2. A CD6 mAb which inhibits T cell activation binds to the top of CD6 domain 1. In SPR analysis, MT605 (A) and MEM98 (B) were injected at 10 µg/ml at 25 ºC, over CD6d1-3S, CD6d1-3S mutants, R77A and E63A or rat CD4 d3+4 in a control flow cell immobilised via OX68 mAb at. Sensorgram traces with response over control flow cell subtracted are shown. Binding of MT605 was specifically reduced by the R77A mutation and not by E63A. There were equivalent levels of all proteins immobilised as monitored by binding of a CD6 domain 3 mAb (OX126).

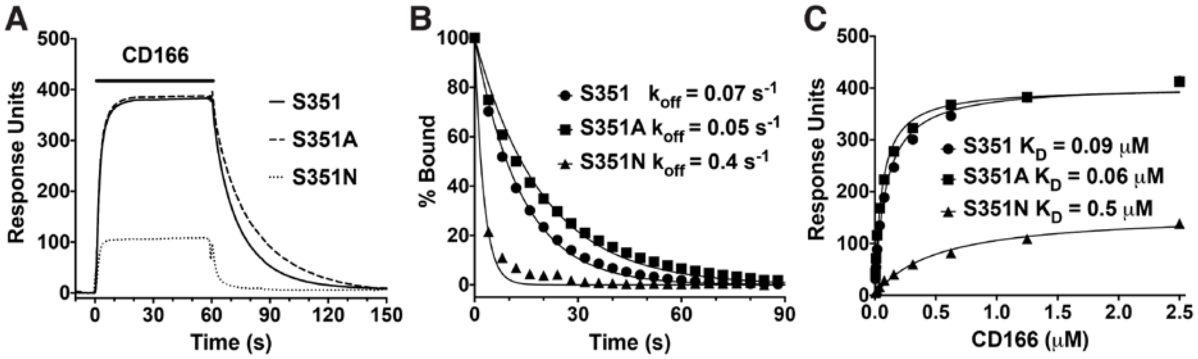

Figure S5, related to Figure 5. A CD6 SNP, S351N in the binding site reduced affinity for CD166. Increasing concentrations of soluble CD166 VVC were injected at 25 °C, over immobilised CD6 S351, CD6 S351A, CD6 S351N and rat CD4 d3+4 in a control flow cell immobilised via OX68 mAb. (A) Sensorgram traces with response over control flow cell subtracted. Bar represents injection period. (B) Dissociation curves for CD166 VVC over immobilised CD6 S351, S351A, or S351N, with response in control flow cell subtracted. The response at t=0 was normalised to 100 % for each flow cell and dissociation monitored over time. The dissociation rate constant, $K_{off}\ s^{-1}$, was calculated for each interaction by non-linear regression using Graphpad Prism (dissociation – one phase exponential decay). (C) Specific binding at equilibrium was calculated by subtracting the response over the control flow cell. The equilibrium dissociation constants, K_D were calculated by curve fitting (Table S4). Top protein concentration: CD166, 2.5 μM; plus nine two-fold serial dilutions were used for the equilibrium binding experiments. There were equivalent levels of all three proteins immobilised as monitored by binding of a CD6 domain 3 mAb (OX126). It is likely that a higher proportion of the immobilised CD6 S351N was inactive leading to a reduction in stoichiometry of binding compared with CD6 S351.

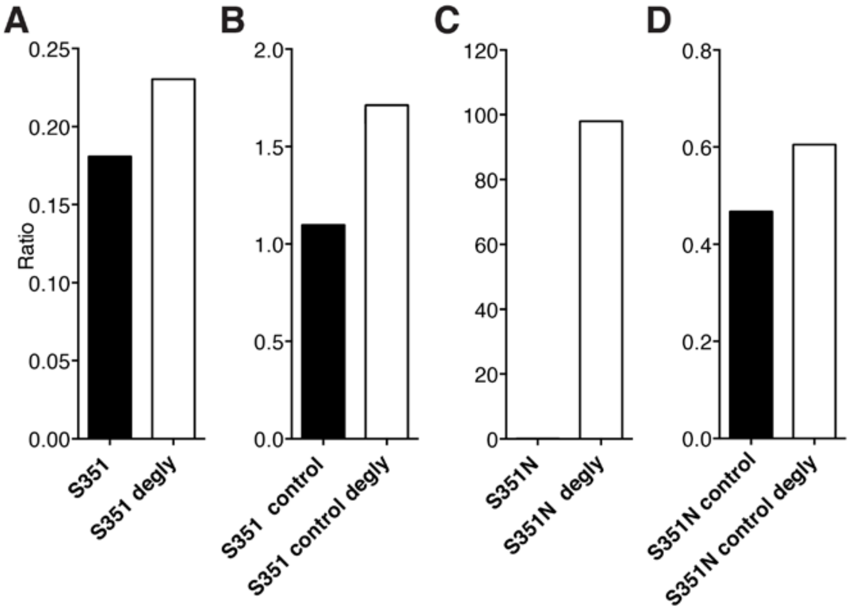

Figure S6, related to Figure 5. The CD6 SNP, S351N introduced an N-linked glycosylation site. Average ratios of the total extracted precursor ion area of peptides from CD6 S351 (A, B) and CD6 S351N (C, D) for S351 (FNNSNLCSQSLAAR) (A), control (B), S351N (FNNSNLCNQSLAAR) (C) and control (D) peptides to precursor ion areas of CD6 control peptides which do not contain N-linked glycosylation sites before (black bars) and after deglycosylation (white bars). The glycosylation site (underlined) at aa 345 (F$\underline{N_{345}NS}$NLCNQSLAAR) in CD6 S351 is not glycosylated.

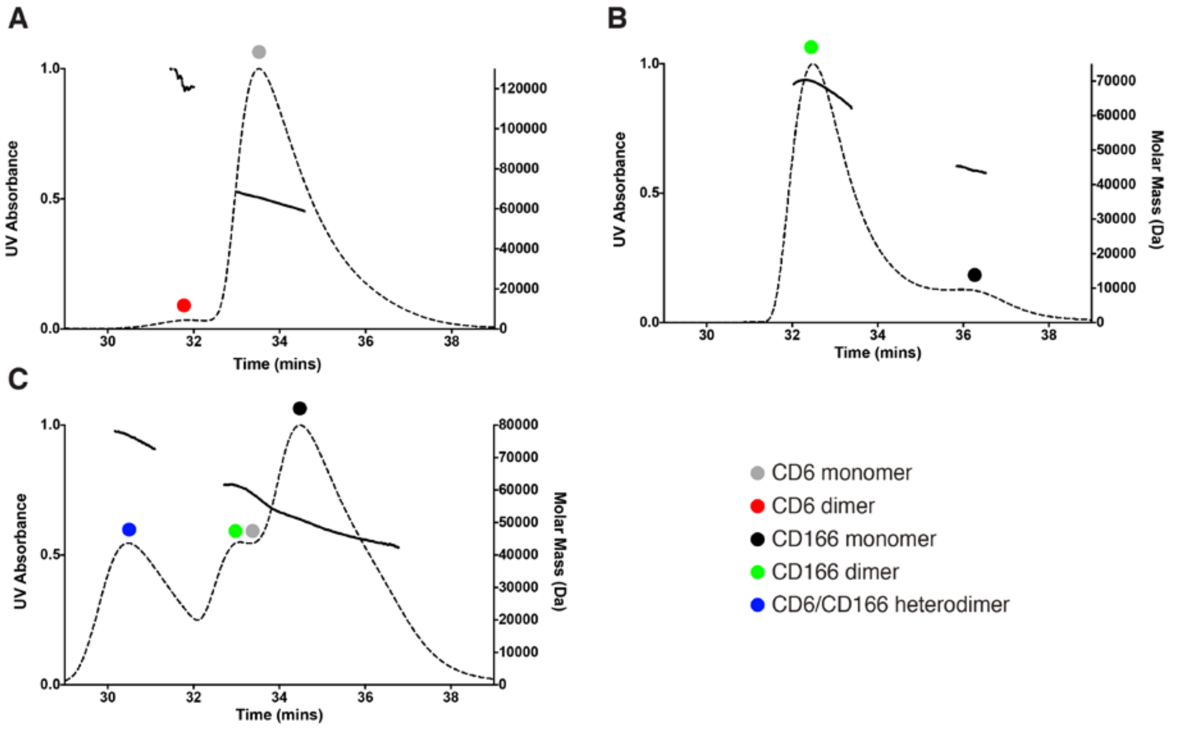

Figure S7, related to Figure 7. MALS showed that CD6 (39 µM) (A) and CD166 VVC (28 µM) (B) are predominantly monomeric and dimeric respectively in solution. Mixing equimolar amounts (27 µM) of CD6 and CD166 VVC (C) altered the species present consistent with formation of heterodimers at the expense of CD6 monomers and CD166 homodimers.

Table S1, related to Figure S2. There is no detectable contribution of CD6 domains 1 and 2 to the interaction with CD166 VVC as measured by SPR at 37ºC. Standard errors of the means were <0.01.

**Molecule immobilised**	**Mean K_D (μM)** ***(range)***	***n***
CD6 d1-3S	0.077 *(0.067 – 0.091)*	3
CD6 d2-3S	0.081 *(0.067 – 0.091)*	3
CD6 d3S	0.086 *(0.077 – 0.097)*	3

Table S2, related to Figures 2 and 5. Residues in CD6 which were mutated to alanine are mapped on the CD6 structure in Figures 2 and 5. (*) = Residues mutated previously to charged residues. For reference, a collated list of the previously reported mutagenesis data is shown (Bodian et al., 1997; Skonier et al., 1997).

Residue	No effect	Disrupted ligand binding	% of WT Binding at K_D ±SEM	*n*
R283*	X		140	1
D291		X	8 ± 4	3
S292		X	37 ± 4	3
E293*		X	15 ± 6	3
Y295		X	4 ± 1	3
E298		X	6 ± 2	3
R314*		X	17 ± 3	3
K316*	X		81 ± 8	3
S323	X		84 ± 13	2
R325	X		88	1
Y327*		X	6 ± 2	3
S329*		X	19 ± 9	3
N331	X		113	1
N346*		X	10 ± 3	3
N348*		X	42 ± 11	3
L349*		X	20 ± 9	3
S351	X		127 ± 6	3
Q352*		X	24 ± 6	3
S353*		X	21 ± 4	3
L354	X		98 ± 23	3

Previously reported mutagenesis data:

Residue	No effect	Disrupted domain structure	Disrupted ligand binding
A271R	X		
Q277R		X	
V285E	X		
W286R		X	
E293R		X	
P296R	X		
Q304R	X		
Q304E	X		
S305R		X	
R314E		X	
S321K	X		
L322R		X	
Y327R		X	
S329R		X	
E333K	X		
N339D	X		
F344R		X	
N345D			X

N346K			X
N348R			X
Q352R			X
S353K		X	
A355D		X	
R357E		X	
S363K		X	

Table S3, related to Figures 2 and 6. Residues in CD166 which were mutated (Skonier et al., 1996a, b) are mapped on the CD166 structure in Figures 2, 5 and 6. (*) = Residues mutated in this study.

Residue	No effect on CD6 binding	Disrupted domain structure	Disrupted CD6 binding	Potential homophilic binding site
Y29A	X			
N32A	X			
I40A		X		
D46A	X			
P48A	X			
N50A	X			
L51A	X			
M52A	X			X
F53A/E			X	X
K55A*/E/D*			X	X
K57A	X			X
E59A	X			
K60A	X			
F67A/E			X	
F70A*/E/Y			X	X
S73A	X			
T74A	X			
K75A/D*			X	
Q79A	X			
Y80A/F	X			
D81A/K*			X	
D82A	X			
E85A	Not expressed			
K87A	X			
R89A	X			
L92A		X		
E94A	X			
S99Y		X		
R104A	X			
R110A	X			
V112A	X			
M114A			X	X
V116Y	X			
T117A			X	
E118A/R*			X	
D119A	X			
N120A	X			
F122A	X			
E123A	X			X
T126Y	X			

Table S4, related to Figure 5 and Figure S6. A CD6 SNP, S351N in the binding site reduced affinity for CD166.

**Molecule immobilised**	**Mean K_D ±SEM (μM)** ***(range)***	**Mean k_{off} ±SEM (s^{-1})** ***(range)***	***n***
S351	0.120 ± 0.016 *(0.089 – 0.144)*	0.072 ± 0.002 *(0.069 – 0.074)*	3
S351N	0.599 ± 0.055 *(0.506 – 0.695)*	0.337 ± 0.030 *(0.280 – 0.384)*	3
S351A	0.083 ± 0.013 *(0.057 – 0.098)*	0.049 ± 0.001 *(0.047 – 0.051)*	3

Table S5, related to Figure 5 and Figure S7. CD6 is glycosylated. [1]Total number of peptide spectral matches (PSM) containing the N glycosylation site before PNGase F treatment. [2]Total number of PSM containing the N glycosylation site after Peptide -N-Glycosidase (PNGase) F treatment. [3]Inconclusive due to low number of peptides containing the N glycosylation site. [4]Although there is an increase of approximately 3 fold more PSM after deglycosylation, there are 43 before, suggesting a partially glycosylated site. [5]Total number of PSM for control peptides containing cysteines before and after deglycosylation.

**Residue**	**N-linked glycosylation site**	**Glycosylation status**	**PSM[1]**	**PSM PNGase F[2]**
28	NTS	YES	1	48
49	NGS	YES	7	91
112	NTS	Inconclusive[3]	0	2
118	NAT	Inconclusive[3]	0	7
229	NCS	YES	1	38
339	NCS	Partial[4]	43	119
345	NNS	NO	162	160
351	NQS	YES	1	80
Cys90[5]	-	-	243	248
Cys170[5]	-	-	51	56

Supplemental Methods Biacore Analysis

Analysis using a BIAcore 3000 was carried out using transiently expressed CD6 and CD166 as fusion proteins with rat CD4 domains 3 and 4 (CD4d3+4) fusion proteins immobilized via a biotinylated tag to streptavidin or rat CD4d3+4 mAb (OX68) (Brown et al., 1998). The CD6 and CD166 fusion proteins were constructed with an XbaI site upstream of the initiation methionine and SalI site (g tcg ac) at the end of the extracellular region in frame with rat CD4d3+4 and all contained the membrane proximal stalk region (Hassan et al., 2004). The same extracellular fragment of CD166 was used to produce soluble CD166d1-5 as a 6 His tagged protein in Lec 3.2.8.1 cells. Similarly to human CD6d3-CD4d3+4, human CD6d2+3-CD4d3+4 was produced from template containing the human CD5 leader (Bowen et al., 1996; Brown et al., 1998). Mutants of CD6 and CD166 were made using a Q5 SDM kit (NEB). K55A, K55D and F70A CD166 mutants were synthesized (Invitrogen). Equilibrium binding and kinetic analyses were carried out in HBS-EP running buffer at 20 µl/min at 37 ºC or 25 ºC as stated in figure legends. Equilibrium binding is plotted after subtraction of the response in the control flow cell and the equilibrium dissociation constant, K_D calculated by curve fitting using a Langmuir 1:1 binding model using Graphpad Prism (one site specific binding). Monoclonal antibodies specific for CD6 domain 1 were MT605 (BD Pharmingen) and MEM98 (abD Serotec), for CD6 domain 3, OX126 (Hassan et al., 2006) and for CD166 (clone 105901, R&D Systems).

Multi-angle Light Scattering (MALS).

Size exclusion chromatography was performed on a Superdex200 10/30 column (GE Healthcare) equilibrated in 50 mM Tris.HCl, pH 7.5, 150 mM NaCl at 0.4 ml/min. The column was followed in-line by a Dawn Heleos-II light scattering detector (Wyatt Technologies) and an Optilab-Rex refractive index monitor (Wyatt Technologies). Molecular mass calculations were performed using ASTRA 5.3.4.14 (Wyatt Technologies) assuming a

dn/dc value of 0.186 ml/g. Samples are diluted approximately 10-fold when applied to the column.

Mass spectrometry analysis of the glycosylation status

CD6-CD4d3+4 S351N transiently expressed in 293T cells was purified with OX68 mAb coupled to Sepharose 4B and elution with 1M Glycine-HCl, pH 2.5. CD6d1-3 His (S351) and CD6-CD4d3+4 S351N, 5-10 µg/sample, were denatured (50mM TCEP-HCl in 8 M urea, 1 h RT), washed with PBS and cysteines alkylated with iodoacetamide (100 µl 20 mM in PBS for 30 minutes at 4 °C). Samples of CD6 S351 and S351N deglycosylated with Peptide -N-Glycosidase (PNGase F) or untreated were digested with trypsin (Metcalfe et al., 2011). After desalting on a C18 micro column, the samples were resuspended in 0.1 % formic acid containing 2 % acetonitrile and analysed on a Ultimate 3000 UHPLC (Dionex) coupled to a QExactive mass spectrometer (Thermo Fisher Scientific). Samples were injected directly on an in-house packed 25 cm C18 (Bishoff 3 µm bead diameter) column in 0.1% formic acid and separated with a gradient of 0.1 % formic acid in acetonitrile, 5-30 % for 90 min, 30 % - 55 % for 20 min and 98 % for 5 min at 300 nl/min. Data were acquired in a data-dependent mode, automatically switching from MS to collision induced dissociation MS/MS on the top 20 most abundant ions with a precursor ion scan range of 350 – 1650 m/z. Full scan MS spectra were acquired at a resolution of 140,000 and MS/MS scans at 17,500 at a target value 3×10^6 and 1×10^5 ions respectively. Dynamic exclusion was enabled with exclusion duration of 40 s.

The data files from all mass spectrometry runs were combined and searched against the human Swiss-Prot database using Peaks 7 proteomics studio. Precursor mass tolerance was 10 ppm and a fragment ion tolerance was 0.01 m/z with up to three missed trypsin cleavage sites per peptide allowed. Variable modifications were defined as deamidation on asparagine and glutamine, oxidation on methionine and carbamidomethylation on cysteine.

De-novo, peaks-db, SPIDER and peaks of post translational modification algorithms were sequentially used to search against a concatenated target/decoy database, providing an empirical false discovery rate (FDR), results are reported at a 1 % target/decoy FDR for both peptides and proteins. 2^+ ions and retention time windows were extracted for the desired peptides (peptide 1 VEMLEHGEWGSVCDDTWDLEDAHVVCR, peptide 2 LVDGGGACAGR, peptide 3 QLGCGWAVQALPGLHFTPGR and peptide 4 GVWNTVCDSEWYPSEAK for non glycosylated control peptides and FNNSNLC(S/N)QSLAAR containing the S351N SNP. Precursor ion areas of the peptides and modified variants such as oxidation of methionine and deamidation were extracted using MS1 filtering in Skyline. The total precursor ion area of each peptide was the sum of all modified variants of that peptide and was used to calculate the following ratios in each sample. 351 peptide:peptide 1, 351 peptide:peptide 2, 351 peptide:peptide 3 and 351 peptide:peptide 4. The following ratios were determined as controls peptide 1:peptide 3, peptide 2:peptide 4, peptide 3:peptide 1 and peptide 4:peptide 2. Averages of the individual 351 and control peptide ratios are reported.

For the other glycosylation sites the total number of peptide spectral matches (PSM) containing each N-glycosylation site before and after deglycosylation with PNGase F were determined from the data produced in the Peaks 7 SPIDER search. As a control the total number of PSM containing Cys90 and Cys70 were determined before and after deglycosylation with PNGase F.

References

Bowen, M.A., Bajorath, J., Siadak, A.W., Modrell, B., Malacko, A.R., Marquardt, H., Nadler, S.G., and Aruffo, A. (1996). The amino-terminal immunoglobulin-like domain of activated leukocyte cell adhesion molecule binds specifically to the membrane-proximal scavenger receptor cysteine-rich domain of CD6 with a 1:1 stoichiometry. J. Biol. Chem. *271*, 17390-17396.

Brown, M.H., Boles, K., van der Merwe, P.A., Kumar, V., Mathew, P.A., and Barclay, A.N. (1998). 2B4, the natural killer and T cell immunoglobulin superfamily surface protein, is a ligand for CD48. J. Exp. Med. *188*, 2083-2090.

Hassan, N.J., Barclay, A.N., and Brown, M.H. (2004). Frontline: Optimal T cell activation requires the engagement of CD6 and CD166. Eur. J. Immunol. *34*, 930-940.

Hassan, N.J., Simmonds, S.J., Clarkson, N.G., Hanrahan, S., Puklavec, M.J., Bomb, M., Barclay, A.N., and Brown, M.H. (2006). CD6 Regulates T-Cell Responses through Activation-Dependent Recruitment of the Positive Regulator SLP-76. Mol. Cell. Biol. *26*, 6727-6738.

Metcalfe, C., Cresswell, P., Ciaccia, L., Thomas, B., and Barclay, A.N. (2011). Labile disulfide bonds are common at the leucocyte cell surface. Open biology *1*, 110010.

Skonier, J.E., Bowen, M.A., Emswiler, J., Aruffo, A., and Bajorath, J. (1996a). Mutational analysis of the CD6 binding site in activated leukocyte cell adhesion molecule. Biochemistry *35*, 14743-14748.

Skonier, J.E., Bowen, M.A., Emswiler, J., Aruffo, A., and Bajorath, J. (1996b). Recognition of diverse proteins by members of the immunoglobulin superfamily: delineation of the receptor binding site in the human CD6 ligand ALCAM. Biochemistry *35*, 12287-12291.
